# Autophagy and Mitophagy in Hypertensive Chronic Kidney Disease: Evidence Grading, Cell-Type Divergence, and a Working Lysosomal Hypothesis

**DOI:** 10.3390/life16091481

**Published:** 2026-09-04

**Authors:** Suyeon Han, Yoon-Kyung Chang, Janghyun Jo, Dae Eun Choi

**Affiliations:** 1 Division of Nephrology, Department of Internal Medicine, College of Medicine, The Catholic University of Korea, Seoul 06591, Republic of Korea; sooyaa1208@catholic.ac.kr (S.H.); racer@catholic.ac.kr (Y.-K.C.); 2 Department of Medical Science, Chungnam National University College of Medicine, Daejeon 35015, Republic of Korea; 3 Department of Nephrology, Chungnam National University Hospital, Daejeon 35015, Republic of Korea

**Keywords:** autophagy, mitophagy, hypertension, chronic kidney disease, lysosome, podocyte

## Abstract

Hypertension is a major attributed cause of chronic kidney disease (CKD) and kidney failure, yet the cellular mechanisms linking chronic hemodynamic and neurohormonal stress to progressive nephron loss remain incompletely defined. Interpretation is further complicated by the clinical heterogeneity and limited pathological validation of hypertensive nephrosclerosis. Autophagy and mitophagy are important intracellular quality-control pathways and have been increasingly implicated in hypertensive kidney injury. In this review, we critically appraise the available evidence using a multidimensional ACGEM framework that evaluates autophagy/mitophagy measurement (A), cell-type resolution (C), genetic manipulation (G), experimental causality (E), and disease-model relevance (M) as independent dimensions. The available literature does not support a uniform increase or decrease in autophagy during hypertensive kidney disease. Rather, autophagic responses appear to depend on renal cell type, hypertensive stimulus, disease stage, and the component of the pathway being measured. In podocytes, chronic angiotensin II exposure provides evidence of impaired autophagic flux with a protective role for intact autophagy, whereas mineralocorticoid stress can induce a compensatory increase in autophagic flux. Tubular studies likewise suggest protective roles for effective autophagic and mitochondrial quality control, although direct cell-specific causal evidence in hypertensive models remains limited. Across the field, most studies rely on static autophagy-associated markers and bulk kidney measurements, while dynamic flux assessment, cell-specific genetic approaches, and direct evaluation of lysosomal competence remain uncommon. Observations from APOL1-associated kidney disease, chronic interstitial nephritis in agricultural communities, proteinuric overload, aging, and obesity provide mechanistic or pathological precedent for lysosomal vulnerability but do not constitute direct evidence for classical hypertensive nephrosclerosis. We therefore propose, as a falsifiable working hypothesis rather than an established mechanism, that lysosomal clearance may become rate limiting in a subset of hypertensive CKD. Testing this model will require longitudinal, cell-type-resolved flux measurements, direct assessment of lysosomal function, and pathological validation in biopsy-confirmed human hypertensive nephrosclerosis.

## 1. Introduction

Chronic kidney disease (CKD) affects more than 10% of the adult population worldwide and represents a major global health burden because of its progressive course, high cardiovascular morbidity and mortality, and risk of kidney failure [1]. Despite substantial etiologic heterogeneity, progressive CKD commonly converges on a shared pathological program characterized by persistent inflammation, maladaptive cellular repair, and kidney fibrosis, with excessive extracellular matrix deposition and progressive disruption of renal architecture [1,2]. Rather than representing a passive end-stage scar, renal fibrosis develops within a dynamic multicellular and molecular microenvironment involving injured epithelial and endothelial cells, fibroblasts and pericytes, infiltrating immune cells, oxidative stress, and profibrotic signaling pathways [1,2,3]. Metabolic remodeling is increasingly recognized as another component of this process: experimental CKD is accompanied by substantial disturbances in phosphatidylcholine metabolism [4], whereas accumulation of bioactive metabolites such as 1-methoxypyrene can promote tubulointerstitial fibrosis through aryl hydrocarbon receptor signaling [5]. Moreover, oxidative stress and inflammation form mutually reinforcing networks that engage pathways including NF-κB, NOX4, Nrf2, TGF-β/Smad, and mitochondrial stress responses, further sustaining renal injury and fibrogenesis [3]. Together, these observations indicate that CKD progression reflects the convergence of hemodynamic, inflammatory, metabolic, and cell-intrinsic stress pathways rather than a single linear mechanism.

Hypertension is particularly important within this framework because its relationship with CKD is bidirectional and self-reinforcing [6]. Declining kidney function impairs sodium and water excretion and promotes volume expansion, while inappropriate intrarenal renin–angiotensin system activation, sympathetic activation, endothelial dysfunction, and inflammatory signaling further contribute to blood-pressure elevation [6]. Conversely, sustained hypertension increases intraglomerular pressure and promotes renal microvascular injury, particularly when autoregulatory protection is impaired, thereby contributing to glomerulosclerosis, tubular ischemic stress, and tubulointerstitial fibrosis [6]. Importantly, hypertensive kidney injury is not solely a consequence of elevated systemic or glomerular pressure. Angiotensin II and other neurohormonal mediators also trigger maladaptive responses within renal parenchymal cells, and recent experimental studies have begun to define these cell-intrinsic mechanisms. In Ang II-induced hypertension, the deubiquitinase OTUD1 promotes epithelial injury, inflammation, and fibrosis through K63-linked deubiquitination and activation of CDK9 [7], whereas CYFIP2 has been implicated in hypertensive renal fibrosis by promoting proximal tubular senescence and dysregulation of p53–Hippo/YAP signaling [8]. These findings support a model in which hypertensive CKD results from the interaction of hemodynamic and vascular stress with maladaptive molecular responses in specific renal cell populations, providing a rationale for examining intracellular quality-control pathways such as autophagy and mitophagy.

Hypertensive nephrosclerosis remains one of the most frequently attributed causes of kidney failure in national and regional registries, although its ranking varies across populations and registries [9]. This label reflects coding practice at registration rather than a mechanistically verified diagnosis. In routine care, nephrosclerosis is usually assigned clinically in patients with hypertension and progressive kidney failure when no alternative explanation is apparent. Biopsy-anchored analyses indicate that these clinical criteria have low sensitivity (approximately 0.13–0.19 with optimized algorithms) and limited discriminatory utility, and that about 40% of clinically labeled cases harbor a different and sometimes treatable diagnosis [10]. In populations of recent African ancestry, *APOL1* high-risk variants are strongly associated with kidney disease clinically attributed to hypertension, supporting the interpretation that a subset of such cases represents *APOL1*-associated glomerular disease rather than kidney injury caused primarily by systemic hypertension [11].

The histopathology attributed to chronic hypertension, namely arteriolar hyalinosis, myointimal thickening, ischemic glomerular obsolescence, focal segmental glomerulosclerosis (FSGS), tubular atrophy, and interstitial fibrosis, suggests chronic, low-grade, sublethal injury to several resident cell populations rather than a single catastrophic insult [12,13]. Cells exposed to such stress depend heavily on intracellular quality-control systems to remove damaged proteins and organelles. Macroautophagy (hereafter autophagy), the lysosome-dependent bulk degradation pathway, and mitophagy, its mitochondria-selective form, are central components of theses quality control systems. Both have been proposed as determinants of whether nephrons adapt to or decompensate under chronic hypertensive stress.

There are strong biological reasons to examine autophagy in hypertensive CKD. Podocytes are terminally differentiated, nonreplicating cells with high constitutive autophagic activity; podocyte-specific deletion of *Atg5* causes accumulation of ubiquitinated protein aggregates, endoplasmic reticulum stress, podocyte loss, proteinuria, and age-dependent glomerulosclerosis [14]. Proximal tubular epithelial cells (PTECs) carry the largest mitochondrial burden in the kidney and depend on oxidative metabolism for solute reabsorption; proximal-tubule-specific *Atg5* deletion leads to accumulation of abnormal mitochondria and cytoplasmic inclusions, cellular hypertrophy, and increased susceptibility to ischemic injury [15]. Both cell types are therefore constitutively autophagy-dependent, and both are injured in hypertensive kidney disease. A mechanistic link between autophagy and hypertensive CKD is biologically plausible before any hypertension-specific experiment is considered.

Interpretive difficulty arises from the types of measurements used to test this link. A large and growing literature reports changes in LC3-II, Beclin-1, ATG proteins, or SQSTM1/p62 in hypertensive kidneys and concludes that autophagy is “activated,” “suppressed,” “impaired,” or “restored.” These conclusions are often based on static measurements in whole-kidney lysates. Autophagy is a dynamic process rather than a static pool: an increase in LC3-II is compatible with both increased autophagosome formation and blocked autophagosome-lysosome fusion, and p62 is a transcriptional target of NFE2L2/NRF2 whose abundance can change independently of degradative flux under oxidative stress [16]. Whole-kidney lysates additionally aggregate signals from podocytes, tubular epithelium, endothelium, fibroblasts, and infiltrating leukocytes, whose autophagic responses may move in different directions. As a result, the same experimental system can support opposite conclusions.

This review does not attempt to combine all reported findings into a single directional narrative of “increased” or “decreased” autophagy. Instead, we evaluate autophagy and mitophagy evidence across separate dimensions of experimental strength, including direct assessment of degradative flux, renal cell-type resolution, genetic manipulation, disease-model relevance, and mechanistic causality. We apply this multidimensional framework across experimental models and major renal cell populations, while distinguishing direct evidence obtained in hypertensive kidney disease from mechanistic evidence derived from diabetic, obstructive, ischemic, or other nonhypertensive kidney injury models. Through this approach, we aim to identify where current evidence is robust, where mechanistic inference remains limited, and which aspects of autophagic and mitochondrial quality control warrant further investigation in hypertensive CKD.

## 2. Methods

A structured literature search was conducted in PubMed from database inception to 19 August 2026. The search strategy and study-selection criteria are summarized in Supplementary Table S1.

Titles and abstracts were screened for relevance, and potentially eligible records underwent full-text assessment. Primary experimental and human studies were eligible when they reported autophagy- or mitophagy-related signaling, markers, degradative flux, or pathway dependence in renal tissue or in defined renal cell types under hypertensive or hypertension-relevant conditions. Nonhypertensive kidney disease models were included only when they addressed a mechanistic question directly relevant to the renal cell type or pathway under discussion, and such studies are identified throughout as contextual rather than direct hypertensive kidney evidence. Reviews, editorials, conference abstracts, and studies without a renal autophagy or mitophagy readout were excluded. Reference lists of relevant primary studies and reviews were screened manually to identify additional records. Fifty-one publications met the inclusion criteria and were carried forward to evidence grading.

Each included publication was graded across the five ACGEM dimensions defined in Table 1: autophagy/mitophagy measurement (A), cell-type resolution (C), genetic evidence (G), causal evidence (E) and model relevance (M). Grading was performed independently by two authors, and discrepancies were resolved by discussion. The unit of analysis was the individual publication: each publication was counted once within each dimension and assigned the highest unequivocally demonstrated grade, and multiple experimental arms, treatment groups or assays within the same publication were not counted as separate studies. Each dimension was graded independently, and no composite score or overall evidence tier was assigned. The complete search strategy and eligibility criteria are provided in Supplementary Table S1, and the study-by-study ACGEM grading of all included publications is provided in Supplementary Table S2.

**Table 1:** Multidimensional framework for evaluating evidence on autophagy and mitophagy in hypertensive kidney disease.

Dimension	Grade	Requirement/Typical Methods	Interpretation
Autophagy/mitophagy measurement (A)	A0	Upstream regulatory signaling only (e.g., mTORC1, AMPK, HIF-1α), without a direct autophagy or mitophagy readout	Provides signaling context only; no conclusion regarding autophagy or mitophagy can be made
	A1	Static autophagy/mitophagy-associated markers or morphology, including LC3-II/LC3-I, BECN1, ATG proteins, SQSTM1/p62, PINK1/PRKN abundance, puncta, colocalization, or electron microscopy	Demonstrates an autophagy- or mitophagy-associated change, but the direction of degradative flux remains unresolved
	A2	Direct dynamic assessment of degradative flux, such as tandem mRFP/mCherry-GFP-LC3 reporters, mt-Keima, mito-QC, or LC3-II turnover under an appropriate lysosomal-inhibitor clamp	Supports a conclusion regarding increased or decreased autophagic or mitophagic degradation
Cell-type resolution (C)	C0	Whole-kidney or bulk-tissue measurements without cellular localization	Cell of origin cannot be determined
	C1	Defined renal cell type in vitro, isolated cell population, or histologic/immunofluorescence localization to a specific renal cell type	Establishes that the observed change is associated with a defined cell type
	C2	Cell-type-specific in vivo readout or manipulation, including conditional genetic models or cell-type-restricted reporters	Establishes in vivo compartment specificity
Genetic evidence (G)	G0	No genetic manipulation of an autophagy- or mitophagy-related component	No genetic evidence for pathway involvement
	G1	Gene knockdown, knockout, overexpression, or genetic rescue in cultured cells or through a non-cell-type-specific in vivo approach	Provides genetic evidence that the manipulated pathway component contributes to the observed response
	G2	Cell-type-specific in vivo deletion, overexpression, or genetic rescue of an autophagy- or mitophagy-related component	Provides strong cell-specific genetic evidence for pathway involvement in vivo
Causal evidence (E)	E0	Descriptive or correlative association between autophagy/mitophagy-related changes and the phenotype	Association only; causal inference is not supported
	E1	Pharmacologic or experimental perturbation modifies both the autophagy/mitophagy readout and the phenotype, but pathway mediation is not definitively established	Supports functional involvement but remains vulnerable to off-target or parallel effects
	E2	Direct manipulation demonstrates that autophagy or mitophagy is necessary for, sufficient to modify, or able to rescue the relevant renal phenotype	Supports causal pathway dependence
Model relevance (M)	M0	Nonhypertensive experimental model or disease context without a defined hypertensive stimulus, such as UUO, cisplatin/ischemic AKI, DKD, or other kidney injury models used primarily for mechanistic comparison	Provides mechanistic context for kidney autophagy/mitophagy but does not constitute direct evidence for hypertensive CKD
	M1	Hypertension-relevant but indirect model, including exposure of renal cells to Ang II, aldosterone, mechanical stretch, or other hypertension-associated stressors in vitro; alternatively, kidney disease models in which hypertension occurs secondarily but is not the defining experimental driver	Supports biological plausibility in a hypertension-relevant context, but extrapolation to hypertensive kidney disease in vivo remains necessary
	M2	Established in vivo hypertensive kidney disease model with documented hypertension and renal injury (e.g., Ang II infusion, DOCA–salt, SHR), or human kidney tissue with sufficiently characterized hypertensive nephropathy, particularly biopsy-anchored disease	Provides direct disease-context evidence relevant to hypertensive CKD

A, autophagy/mitophagy measurement; C, cell-type resolution; G, genetic evidence; E, causal evidence; M, model relevance. Each dimension is assessed independently, with the highest unequivocally demonstrated category reported; no composite score or overall evidence tier is assigned. M reflects disease-context relevance and does not alter the A, C, G, or E grades. Static changes in LC3, SQSTM1/p62, BECN1, PINK1, PRKN, or related proteins are not considered evidence of altered degradative flux without an appropriate dynamic flux assay.

## 3. Autophagy and Mitophagy in Kidney Cells: Machinery, Regulation, and Measurement

### 3.1. Core Machinery

Macroautophagy proceeds through initiation, nucleation, elongation, closure, lysosomal fusion, and degradation. The ULK1 complex (ULK1, ATG13, RB1CC1/FIP200, ATG101) initiates the phagophore. The class III phosphatidylinositol 3-kinase complex containing BECN1/Beclin-1, PIK3C3/VPS34, and ATG14 nucleates the isolation membrane. Two ubiquitin-like conjugation systems, ATG12-ATG5-ATG16L1 and the ATG8/LC3 lipidation cascade requiring ATG7 and ATG3, drive elongation and closure [16].

Lipidated LC3-II remains on autophagosome membranes and underpins most commonly used assays. SQSTM1/p62 links ubiquitinated cargo to LC3 and is degraded within autolysosomes, so its accumulation is conventionally read as impaired degradation. Fusion with lysosomes, mediated by RAB7, HOPS-complex components, and SNAREs, delivers cargo to hydrolases [17]. Transcriptional control of autophagic and lysosomal genes is coordinated by TFEB [18].

Mitophagy is the selective delivery of mitochondria to this pathway. The best-characterized route is the PINK1-PRKN/Parkin axis, in which loss of mitochondrial membrane potential stabilizes PINK1, recruits and activates PRKN, and generates phospho-ubiquitin chains recognized by autophagy receptors [19]. Receptor-mediated routes independent of PRKN also exist, including BNIP3, BNIP3L/NIX, FUNDC1, and PHB2 [20,21,22,23].

This distinction is important in the kidney. Reporter-mouse studies show that renal tubules are among the most active sites of basal mitophagy in the mammalian body and that basal mitophagy in high-demand tissues occurs largely independently of PINK1 [24,25]. Nonetheless, several studies of hypertensive kidney injury have interpreted changes in PINK1 and PRKN abundance as evidence of altered mitophagy, although direct measurements of mitophagic flux remain unavailable [26,27,28].

### 3.2. Regulatory Inputs Converging on Hypertensive Signaling

Several signaling axes that are pathologically engaged in hypertensive kidney disease are core regulators of autophagy, which provides a mechanistic basis to expect interaction.

mTORC1. mTORC1 phosphorylates ULK1 and TFEB and is the principal negative regulator of autophagy initiation. In Dahl salt-sensitive rats, high salt activates renal mTORC1, and pharmacologic mTORC1 inhibition with rapamycin attenuates salt-induced hypertension and kidney injury [29]. Subsequent work linked these effects to NOX4-derived hydrogen peroxide: *Nox4* deletion reduced mTORC1 activation, hypertension, and renal injury, and rapamycin conferred no further protection in *Nox4*-null rats [30]. These data support an oxidative stress-mTORC1 axis in salt-sensitive hypertension but do not directly measure autophagy; the inference that autophagy is impaired in this setting remains a hypothesis.

AMPK and SIRT1-FOXO signaling. Energy stress activates AMPK, which phosphorylates ULK1 at activating sites and inhibits mTORC1. AMPK-ULK1 signaling has been directly implicated in protective epithelial autophagy under mineralocorticoid stress [31]. SIRT1, an NAD+-dependent deacetylase, can regulate autophagy through deacetylation of FOXO transcription factors and core ATG proteins [32,33]. In hypertensive-nephropathy intervention studies, however, involvement of this pathway has been inferred primarily from changes in SIRT1–FOXO3a or SIRT1–PGC-1α signaling together with static measurements of LC3, p62, Beclin-1, PINK1, and PRKN; direct in vivo measurements of autophagic or mitophagic flux remain lacking [26,34].

Hypoxia and HIF-1α. Peritubular capillary rarefaction and interstitial fibrosis increase oxygen diffusion distance in hypertensive kidneys [35]. HIF-1α induces BNIP3 and BNIP3L, providing a route to receptor-mediated mitophagy [36]. Sustained HIF-1α signaling has also been associated with profibrotic, autophagy-related responses in hypertensive models, so its net effect is context-dependent [37].

Mechanical stress and mineralocorticoid receptor signaling. Glomerular capillary hypertension imposes cyclic tensile stress on podocytes [38,39]. In cultured human podocytes, prolonged stretch activates PI3K-AKT-mTOR signaling and reduces LC3 puncta and ATG5 abundance, and mineralocorticoid receptor (MR) or PI3K/mTOR blockade partially reverses these effects [40]. Mechanical load and MR signaling therefore converge on the regulatory node that controls autophagy initiation.

### 3.3. Why Static Markers Mislead: A Multidimensional Framework for Evidence Assessment

Interpretive difficulty in this literature arises less from experimental quality than from the type of measurement used. Most studies use standard techniques and are technically sound. The difficulty is that static abundance of autophagy-related proteins in bulk tissue is frequently asked to support conclusions about flux, direction, and causality that it cannot, on its own, sustain.

Three recurring problems limit interpretation. First, directional ambiguity: LC3-II abundance reflects the balance between autophagosome formation and clearance, and without tandem reporters or lysosomal-inhibitor clamps an increase cannot be assigned to either [16]. Second, marker nonspecificity: BECN1 participates in nonautophagic trafficking, ATG5 and ATG7 have roles beyond autophagy, and SQSTM1/p62 is transcriptionally induced by NFE2L2/NRF2 under oxidative stress [41,42,43]. A rise in p62 in an angiotensin II (Ang II)-infused kidney is therefore not, by itself, evidence of blocked degradation [44]. Third, compartment aggregation: whole-kidney lysates mix signals from glomeruli, tubules, vessels, interstitium, and immune cells and cannot distinguish opposing changes between compartments.

To address these interpretive limitations, we evaluated the strength of autophagy and mitophagy evidence across multiple independent methodological dimensions rather than relying on any single measure of experimental strength. These dimensions capture distinct aspects of study design, including direct assessment of degradative flux, cell-type resolution, genetic manipulation, and causal pathway dependence, which provide complementary rather than interchangeable information. For example, one study may directly measure autophagic flux without resolving the responsible renal cell type, whereas another may employ a cell-type-specific genetic model without directly measuring degradative flux. Evaluating these features independently therefore allows the strengths and limitations of each experimental design to be represented more transparently.

Accordingly, we used a multidimensional evidence assessment framework comprising five independent domains: autophagy/mitophagy measurement (A), cell-type resolution (C), genetic evidence (G), causal evidence (E), and model relevance (M), hereafter referred to as the ACGEM framework (Table 1). The A domain evaluates how directly autophagic or mitophagic activity is measured, ranging from inference based on upstream signaling to static pathway-associated markers and direct assessment of degradative flux. The C domain evaluates the anatomical and cellular resolution of the evidence, distinguishing bulk kidney measurements from studies in defined renal cell populations and from cell-type-specific in vivo approaches. The G domain records whether autophagy- or mitophagy-related genes are directly manipulated and whether such manipulation is performed in a cell-type-specific manner. The E domain evaluates the strength of mechanistic inference supported by the experimental design, distinguishing descriptive associations from functional perturbation and from experiments demonstrating pathway necessity, sufficiency, reversal, or rescue. The M domain evaluates the directness of the experimental context to hypertensive kidney disease, distinguishing established in vivo hypertensive models from hypertension-relevant cellular stress models and nonhypertensive kidney disease models included for mechanistic context.

**Figure 1:**
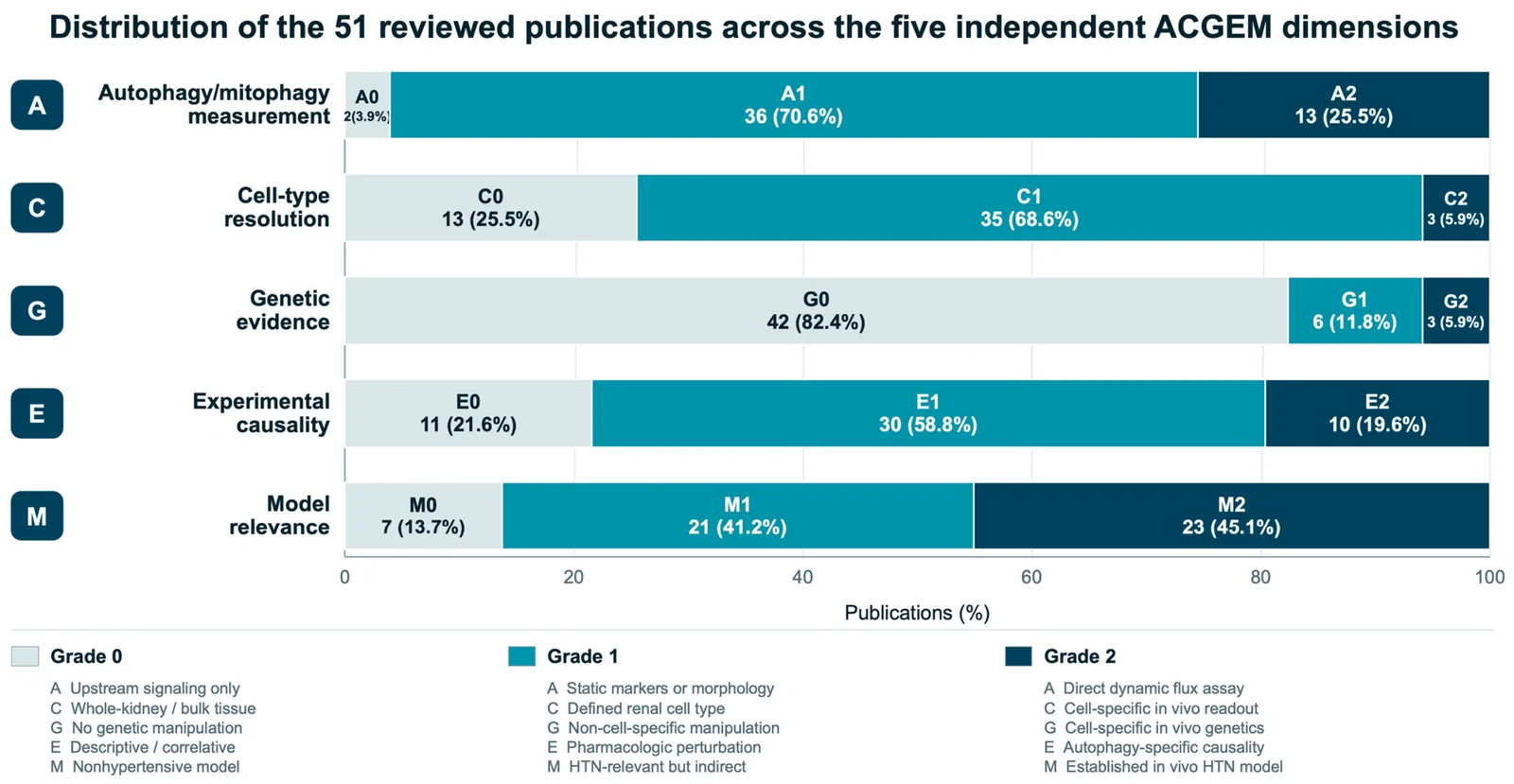
Distribution of the reviewed publications across the independent ACGEM evidence dimensions. The 51 publications included in the study-by-study assessment were graded independently according to five dimensions: A, autophagy/mitophagy measurement; C, cell-type resolution; G, genetic evidence; E, experimental causality; and M, model relevance. Each dimension was graded from 0 to 2 according to Table 1, without calculation of a composite score or overall evidence tier. The unit of analysis was the individual publication, and values within the bars indicate the number and percentage of publications assigned to each grade. Most studies relied on static autophagy-associated measurements (A1, 70.6%) and had limited cell-type resolution (C1, 68.6%), while cell-specific in vivo approaches and genetic evidence were uncommon (C2 and G2, each 5.9%). In contrast, disease-model relevance was relatively strong, with 45.1% of studies graded M2. These distributions highlight that high relevance to hypertensive kidney disease does not necessarily imply strong mechanistic evidence for altered autophagy or mitophagy, supporting independent evaluation of the ACGEM dimensions. The study-by-study ACGEM grades underlying this figure, together with the experimental model, renal compartment or cell type, principal autophagy/mitophagy readouts and key limitations of each publication, are provided in Supplementary Table S2.

Each dimension is evaluated independently, and no composite score or overall evidence tier is assigned. Thus, strength in one dimension does not imply strength in another. In particular, model relevance (M) reflects disease-context applicability rather than methodological rigor and does not modify the A, C, G, or E grades. A study performed in a nonhypertensive model may therefore provide strong flux, cell-specific, genetic, or causal evidence while remaining indirect with respect to hypertensive CKD; conversely, a study using a well-established hypertensive model may have high model relevance despite limited autophagy/mitophagy measurement or cell-type resolution. Where relevant, study provenance is noted separately to identify findings arising from overlapping investigative groups or institutional lineages and to avoid interpreting related reports as fully independent replication. This multidimensional approach preserves the specific strengths and limitations of each experimental design without collapsing distinct forms of evidence into a single linear judgment. The distribution of ACGEM grades across the 51 included publications is summarized in Figure 1.

## 4. Which Stresses Reach Which Cells: The Pathophysiologic Basis for Cell-Type-Specific Analysis

Hypertensive kidney injury is often described as a uniform response to a single stress, namely elevated pressure. It is not. Pressure, neurohormonal signaling, filtered protein load, hypoxia, and mitochondrial damage reach different cell types at different disease stages. This heterogeneity is what makes cell-type-resolved analysis necessary, and it directly shapes how autophagy should be interpreted in each compartment.

### 4.1. Mechanical Loading and Impaired Autoregulation

In health, myogenic and tubuloglomerular feedback attenuate transmission of systemic pressure to the glomerular capillary bed. When preglomerular autoregulation fails, systemic pressure reaches the glomerulus, imposing tensile and shear stress on podocytes and endothelium [45]. Podocytes are postmitotic and adherent to the glomerular basement membrane through an elaborate foot-process architecture; they cannot withstand sustained mechanical loading and respond by hypertrophy, foot-process effacement, and detachment [46,47]. This mechanical stress falls almost entirely on the glomerulus and increases the reliance of podocytes on autophagic quality control [14].

### 4.2. Neurohormonal Signaling

Intrarenal Ang II acts through AT_1_ receptors on podocytes, tubular epithelium, mesangial cells, endothelium, and infiltrating immune cells, generating NADPH oxidase-dependent reactive oxygen species (ROS), mitochondrial dysfunction, NF-κB activation, and TGF-β-driven fibrosis [48,49,50,51,52]. Aldosterone acting on the MR produces an overlapping but distinguishable program, with SGK1 acting as a downstream effector and Rac1 serving as an important amplifier, and in some settings a ligand-independent activator, of MR signaling [53,54]. Because both ligands act directly on cells while also raising pressure, no model driven by them isolates purely pressure-mediated injury, a point that becomes important in Section 5.

### 4.3. Filtered Protein Load

Once the glomerular barrier is compromised, PTECs endocytose an increased filtered protein burden that must be processed lysosomally. Pathologic albumin exposure suppresses autophagic flux in proximal tubular cells through mTOR-dependent signaling [55]. Proteinuria therefore acts as a secondary amplifier: glomerular injury generates a tubular quality-control stress that did not exist at the outset, giving hypertensive CKD an intrinsically staged structure.

### 4.4. Chronic Hypoxia

Peritubular capillary rarefaction and interstitial fibrosis reduce oxygen delivery to the most metabolically demanding compartment of the kidney [35]. The result is impaired oxidative phosphorylation and mitochondrial redox stress, precisely the conditions that generate mitophagy substrate in the tubular compartment.

### 4.5. Mitochondrial Damage as a Signaling Event

When mitochondrial integrity is lost, mitochondrial DNA (mtDNA) enters the cytosol and activates innate immune signaling. In human hypertensive nephropathy and in Ang II and two-kidney one-clip (2K1C) models, tubular mitochondrial disruption, cytosolic mtDNA accumulation, and STING activation have been demonstrated, and epithelial-specific STING deletion reduces inflammation and fibrosis [56]. Urinary mtDNA copy number is also increased in patients with essential and renovascular hypertension and correlates with renal injury [57]. Mitochondrial damage in HTN-CKD is therefore not only a metabolic event but a driver of inflammation.

### 4.6. Stage- and Cell-Specific Patterns of Stress

The considerations in Section 4.1, Section 4.2, Section 4.3, Section 4.4 and Section 4.5 have two consequences for how autophagy should be interpreted in hypertensive kidney disease. First, the stresses are staged: mechanical and neurohormonal stress precede proteinuric and hypoxic stress, so the autophagic state of a hypertensive kidney should be expected to vary with disease stage [58,59,60]. Second, they are cell-specific: podocytes experience predominantly mechanical and neurohormonal stress, whereas PTECs experience predominantly metabolic, proteinuric, and hypoxic stress [58,59,60]. There is no a priori reason for autophagy to change in the same direction in both compartments, and Section 6 and Section 7 confirm that it does not.

A separate point concerns the direction of causality. Autophagy is usually treated as a downstream consequence of hypertension. However, renal and vascular autophagy also participate in blood pressure regulation itself, through effects on tubular sodium handling, endothelial function, and immune cell activation. Interventions that modify renal autophagy while also lowering blood pressure cannot separate renoprotection mediated by autophagy from renoprotection mediated by pressure reduction. This confounder is discussed further in Section 10.1.

## 5. Experimental Models of Hypertensive Kidney Disease and Their Consequences for Autophagy Research

Experimental models of hypertension reproduce distinct components of hypertensive kidney disease, including elevated systemic pressure, RAAS activation, mineralocorticoid excess, salt loading, and renal ischemia, but no single model recapitulates the slowly progressive vascular, glomerular, and tubulointerstitial pathology of human hypertensive CKD [61,62] Systematic comparative pathology has made this concrete: across commonly used hypertensive models, the severity and distribution of glomerular and tubular lesions differ substantially from human hypertensive nephrosclerosis, and several models produce markedly milder disease than the human condition they are taken to represent [12,13].

This heterogeneity is not merely a peripheral caveat but a major determinant of autophagy readouts. Autophagic responses are shaped by the initiating stimulus, the duration and severity of hypertension, the metabolic environment, and the cell type examined. Discrepancies between models should therefore be interpreted as information about context dependence rather than as contradictions requiring resolution.

### 5.1. Angiotensin II Infusion

Chronic Ang II infusion, typically delivered by osmotic minipump over 2–4 weeks, is the most widely used model in this field [62]. It combines systemic hypertension with direct intrarenal AT1 receptor signaling, oxidative stress, inflammation, mitochondrial dysfunction, and fibrosis, making it difficult to separate pressure-mediated injury from direct Ang II effects [62].

Autophagy findings are inconsistent. Shen et al. reported increased renal BECN1 and LC3-II/LC3-I after 2 weeks of Ang II infusion, whereas Majumder et al. found reduced ATG3, ATG12, BECN1, and LC3 with increased SQSTM1/p62 after 4 weeks [63,64]. These studies should not be interpreted as a simple early-activation-to-late-failure sequence because they differ in experimental design and neither directly measured autophagic flux. More recently, daily subcutaneous Ang II administration for 4 weeks increased LC3-II/LC3-I and ATG3/5/7 while reducing p62; treadmill exercise attenuated these changes together with renal injury and fibrosis [65]. However, these were again whole-kidney static measurements without lysosomal inhibition, so neither flux direction nor cell-specific mechanisms could be established.

Overall, Ang II models are useful for examining RAAS–autophagy interactions, but autophagy-related protein abundance appears highly context dependent and cannot be equated with degradative flux. In addition, chronic Ang II exposure does not fully reproduce the glomerulosclerosis and tubulointerstitial pathology of human hypertensive nephrosclerosis, limiting its fidelity as a model of pressure-mediated hypertensive CKD [12,13].

### 5.2. Spontaneously Hypertensive Rats

The spontaneously hypertensive rat (SHR) develops genetically determined hypertension without exogenous vasoactive hormone administration, making it attractive for studying long-term adaptation and interaction with aging, diet, and exercise. Autophagy findings are again heterogeneous. Jia et al. showed that a ketogenic diet further activated the RAAS, reduced renal autophagy-related protein expression, and aggravated renal injury in SHRs [66]. Li et al. reported the converse pattern, namely increased renal HIF-1α, ATG5, and LC3B-II with decreased p62 in sedentary SHRs with fibrosis, and found that moderate-intensity continuous training reduced HIF-1α, these autophagy-related changes, and fibrosis together [37].

An important structural limitation of the SHR is that hypertension does not reliably translate into advanced nephrosclerosis. Conventional SHRs, particularly at younger ages, develop sustained hypertension with comparatively mild glomerular and tubulointerstitial lesions, and comparative studies found little evidence of the characteristic glomerulosclerosis or hypertensive tubulopathy of advanced human disease [12,13]. Renal autophagy changes in standard SHRs may therefore reflect cellular adaptation to chronic hypertension rather than mechanisms of established hypertensive CKD.

### 5.3. Mineralocorticoid-Salt Hypertension

Deoxycorticosterone acetate (DOCA)-salt and aldosterone-salt models emphasize MR activation, sodium excess, and volume-dependent hypertension, typically with suppressed systemic renin activity. They therefore provide a complementary system in which MR- and salt-dependent injury can be examined without high circulating Ang II. Renal injury in this model is not attributable to pressure alone: Klanke et al. showed that both pressure-dependent mechanisms and direct mineralocorticoid effects contribute to renal inflammation and fibrosis [67]. These models have generated some of the strongest functional evidence linking autophagy to tubular injury in experimental hypertension. Li et al. found that AdipoRon attenuated renal fibrosis in DOCA-salt hypertensive mice while promoting autophagy. In aldosterone-treated HK-2 cells, AdipoRon activated AMPK–ULK1 signaling and increased autophagic flux, as demonstrated using tandem mRFP-GFP-LC3, whereas inhibition of AMPK or autophagy attenuated its anti-EMT and antifibrotic effects [31]. Accordingly, this study was classified as A2/C1/G0/E1/M2, combining disease-relevant hypertension with dynamic flux assessment and pharmacological pathway-dependence testing, although flux was demonstrated in an immortalized tubular cell line rather than directly in hypertensive kidneys.

Complementary in vivo evidence was provided by Zhang et al. in aldosterone-salt hypertensive rats [68]. Pharmacological inhibition of GSK-3β with SB-216763 reduced renal inflammation, fibrosis, and tubulointerstitial injury while increasing LC3-II and LC3-positive puncta and decreasing p62 [68]. Importantly, 3-MA partially reversed the renoprotective effect of SB-216763, supporting a functional contribution of autophagy to mineralocorticoid-associated renal injury [68]. However, autophagic flux was not directly measured and the autophagy signal was not localized to a specific renal cell population; moreover, SB-216763 also lowered blood pressure, making it difficult to separate autophagy-dependent protection from reduced hemodynamic injury. This study was therefore classified as A1/C0/G0/E1/M2.

Together, these studies support a protective role for productive autophagy in mineralocorticoid-salt injury, but they also illustrate the distinction between disease relevance and autophagy-specific evidence. The AdipoRon study provides stronger evidence for tubular autophagic flux, whereas the SB-216763 study provides broader in vivo functional support without cell-type resolution. The supraphysiological mineralocorticoid and salt loading used in these models should also be considered when extrapolating these findings to essential hypertension.

### 5.4. Renovascular Hypertension: The Two-Kidney One-Clip Model

The Goldblatt 2K1C model generates hypertension by unilateral renal artery stenosis, so that RAAS activation arises endogenously from reduced renal perfusion [69]. Its distinguishing feature, and its principal analytical difficulty, is that the two kidneys sustain fundamentally different insults [70,71]. The clipped kidney experiences chronic hypoperfusion and ischemic stress, whereas the nonclipped kidney experiences systemic hypertension, circulating RAAS activation, and compensatory hyperfiltration [70,71]. Pooled or whole-animal analyses therefore average two distinct pathologies [71]. Comparative studies found relatively mild hypertensive lesions in the nonclipped kidney and more severe tubulointerstitial pathology in the clipped kidney, the latter inseparable from ischemia [12,13].

Direct autophagy or mitophagy data in this model are limited. Its main contribution has been to show that mitochondrial injury and innate immune activation are shared across forms of experimental hypertension: Gao et al. demonstrated mitochondrial disruption, cytosolic mtDNA accumulation, and STING activation in both Ang II and 2K1C models [56]. Autophagic and mitophagic flux were not assessed, so this work demonstrates a consequence of compromised mitochondrial integrity rather than a defect in mitochondrial turnover.

### 5.5. Dahl Salt-Sensitive Rat Model

The Dahl salt-sensitive (Dahl SS) rat is a well-established model of salt-sensitive hypertension and hypertensive kidney injury. As discussed in Section 3.2, high-salt feeding activates renal mTORC1 through oxidative stress-related mechanisms, and mTORC1 inhibition attenuates hypertension and renal injury [29,30]. However, these studies did not directly assess autophagy, precluding inference regarding autophagic activity from mTORC1 signaling alone.

Direct, although still limited, autophagy-related evidence was provided by Takakura et al. using Dahl SS rats exposed to a high-salt diet [72]. The kidneys showed structural injury involving both glomerular podocytes and tubular epithelial cells, whereas *Cordyceps militaris* treatment ameliorated these abnormalities. Treatment was also associated with increased renal LC3-II/LC3-I and cathepsin D and decreased p62, together with changes in mitochondrial proteins, which the authors interpreted as enhancement of autophagy and mitochondrial function [72]. However, these measurements were based on static protein abundance rather than dynamic flux, and no autophagy-specific perturbation was performed, so renal protection cannot be causally attributed to autophagy on this basis. Treatment did not significantly lower blood pressure, which makes a purely pressure-mediated benefit less likely but does not in itself support an autophagy-dependent mechanism.

More recent proteomic evidence provides a closer, although still indirect, link to the autophagy machinery. In Dahl SS rats with partial Mlycd deficiency, bulk-kidney proteomics showed enrichment of autophagy- and mTOR-related pathways and reduced abundance of Vps34/Pik3c3, Atg9, and Vps16, implicating alterations in both autophagosome formation and autophagosome–lysosome fusion [73]. Nevertheless, autophagic flux was not measured, and Mlycd deficiency did not further increase blood pressure during high-salt challenge. Thus, these findings indicate remodeling of autophagy-associated machinery in a hypertension-prone background rather than hypertension-induced impairment of autophagic degradation. Overall, the Dahl SS model provides strong evidence for renal metabolic and mTORC1 dysregulation during salt-sensitive hypertension, but direct evidence defining the direction and functional consequences of autophagic flux remains limited.

### 5.6. Models of Reduced Renal Mass and Nitric Oxide Deficiency

Subtotal (5/6) nephrectomy causes substantial nephron loss followed by compensatory glomerular hyperfiltration, proteinuria, progressive fibrosis, and secondary hypertension. Although widely used to model CKD progression, it is only indirectly relevant to HTN-CKD because renal mass reduction is the primary insult and hypertension develops subsequently [74].

Within this model, autophagy findings vary according to experimental design. Matsui et al. provided relatively strong mechanistic evidence by using in vivo flux assessment and inducible proximal-tubule-specific *Atg5* manipulation, showing that chronic metabolic and nephron-reduction stress can exceed tubular autophagic–lysosomal capacity [75]. However, because the initiating insult was reduced nephron mass rather than hypertension, this study is best regarded as mechanistic support for a clearance-limited autophagy phenotype rather than direct evidence for hypertensive kidney disease. In contrast, Li et al. reported that HeidihuangWan improved renal fibrosis after 5/6 nephrectomy while reducing BECN1 and LC3-II through IGF-1/PI3K/Akt/mTOR signaling; however, these conclusions were based on static whole-kidney autophagy markers and did not establish autophagic flux [76]. A more hypertension-relevant modification was used by Ko et al., who combined 5/6 nephrectomy with DOCA-salt and showed improvement in renal injury accompanied by changes in LC3B, ATG5, BECN1, and PINK1/PARKIN after pharmacologic treatment [77]. Thus, reduced-renal-mass models provide useful mechanistic context for autophagy and mitophagy in chronic renal stress, but evidence from nephrectomy alone should remain secondary to studies in which hypertension is the primary driver of kidney injury.

Chronic nitric oxide synthase inhibition with N^ω^-nitro-L-arginine methyl ester (L-NAME) produces endothelial dysfunction and hypertension but has generated very little direct renal autophagy data, and is not a productive platform for this question at present.

### 5.7. Implications of Model Heterogeneity

Table 2 summarizes the models against the phenotypes they reproduce and the strength of available autophagy evidence.

Two conclusions follow. First, the models emphasize distinct pathogenic mechanisms, so divergent autophagy findings across them are expected rather than anomalous. Conclusions about autophagy in HTN-CKD should therefore be based on convergence across complementary models, with disease stage, renal compartment, and cell type explicitly considered. Second, the ACGEM assessment reveals a marked imbalance across evidence dimensions: although several models have strong relevance to hypertensive kidney injury, direct assessment of autophagic flux, cell-type-resolved in vivo analysis, genetic manipulation of the autophagy machinery, and autophagy-specific perturbation remain limited. Much of the literature relies on static autophagy-associated markers in whole-kidney tissue, while some highly relevant models provide only signaling context without directly measuring autophagy. These limitations provide the rationale for the cell-type-resolved analysis that follows.

## 6. Podocyte Autophagy in Hypertensive Kidney Disease

### 6.1. Basal Autophagy and Podocyte Homeostasis

Podocytes exhibit high constitutive autophagic activity, consistent with their postmitotic state and specialized cytoskeletal architecture. Podocyte-specific *Atg5* deletion causes accumulation of ubiquitinated and oxidized proteins, endoplasmic reticulum stress, podocyte loss, proteinuria, and age-dependent glomerulosclerosis, and increases susceptibility to experimental glomerular injury [14]. Endothelial and podocyte autophagy also act synergistically to protect the glomerular filtration barrier in metabolic disease [80].

These findings define the baseline against which hypertensive injury must be read. Intact autophagic quality control is not merely a secondary response but a precondition for podocyte tolerance of sustained stress.

### 6.2. Angiotensin II and Impairment of Podocyte Autophagic Flux

Acute Ang II exposure consistently alters autophagy-associated pathways in cultured podocytes, although most studies have relied on static markers rather than direct measurements of autophagic flux. Yadav et al. reported increased LC3 and autophagosome abundance associated with ROS generation after Ang II exposure (A1/C1/G0/E0/M1) [81]. Subsequent pharmacologic studies similarly linked Ang II-induced ROS to changes in autophagy-related markers. Sinomenine reduced LC3B-associated structures and autophagosome/autolysosome abundance together with inhibition of NADPH oxidase p47-phox membrane translocation (A1/C1/G0/E0/M1) [82], whereas ginsenoside Rg1 suppressed Ang II-associated LC3-II and BECN1 changes through AMPK/GSK3β/mTOR-P70S6K signaling (A1/C1/G0/E0/M1) [83]. Because neither study directly measured dynamic flux, reduced autophagosome abundance cannot distinguish reduced autophagosome formation from altered downstream clearance.

Other short-term experiments suggest that the autophagy-associated response may initially be adaptive. Gao et al. linked ROS-dependent Ca^2+^ signaling to increased LC3-II/I and BECN1, with pharmacologic enhancement or inhibition of autophagy modifying podocyte viability (A1/C1/G0/E1/M1) [84]. Seong et al. similarly found that autophagy-associated changes preceded apoptosis after Ang II exposure and that *Atg5* knockdown increased susceptibility to injury (A1/C1/G1/E2/M1) [85]. Collectively, these experiments support an early stress-associated autophagic response to Ang II, but the absence of dynamic flux measurements prevents firm conclusions regarding the direction of autophagic activity.

Evidence from disease-relevant therapeutic studies remains similarly indirect. In spontaneously hypertensive rats and Ang II-treated podocytes, Qian Yang Yu Yin granules reduced autophagy-associated markers together with podocyte injury through TRPC6–CaMKKβ–AMPK–mTOR-associated signaling (A1/C1/G0/E1/M2) [86]. More recently, Yanggan-Yishui granules reversed Ang II-associated changes in autophagosome abundance, LC3-II/I, p62, and BECN1 through PI3K/AKT/mTOR-associated signaling in MPC-5 cells (A1/C1/G0/E1/M1) [87]. These studies demonstrate modulation of autophagy-associated machinery but do not establish whether changes in autophagy mediate the observed protection.

In contrast, chronic Ang II exposure provides substantially stronger evidence for impaired autophagic flux. Bensaada et al. demonstrated impaired glomerular autophagic flux during chronic Ang II-induced hypertension and showed that podocyte-specific *Atg5* deletion aggravated podocytopathy, albuminuria, and glomerulosclerosis [78]. Ang II activated calpain, whereas transgenic overexpression of its endogenous inhibitor calpastatin preserved autophagy and attenuated podocyte injury and albuminuria. This study provides the strongest podocyte evidence currently available in hypertensive kidney disease (A2/C2/G2/E2/M2), combining dynamic flux assessment, podocyte-specific genetic manipulation, functional renal outcomes, and an in vivo hypertensive model [78].

Taken together, the available evidence supports a context- and duration-dependent model rather than a simple directional effect of Ang II on podocyte autophagy. Acute Ang II exposure is associated with autophagy-related marker changes that may represent an adaptive stress response, whereas chronic Ang II-induced hypertension can impair autophagic flux through the calpain–calpastatin axis. However, this temporal model remains an inference across separate experimental systems rather than the result of a longitudinal study directly measuring podocyte autophagic flux from early to chronic Ang II exposure.

### 6.3. Aldosterone and MR Signaling and Compensatory Autophagy

Aldosterone/MR signaling injures podocytes through oxidative stress, endoplasmic reticulum stress, mitochondrial dysfunction, and loss of slit-diaphragm proteins [53,88]. In contrast to the impaired flux demonstrated during chronic Ang II exposure, several studies suggest that aldosterone initially induces an adaptive autophagic response.

Mao et al. reported increased LC3-II, BECN1, and autophagosome abundance in aldosterone-treated MPC5 podocytes, with chloroquine-supported LC3 accumulation providing evidence consistent with increased autophagic activity (A2/C1/G0/E0/M1) [89]. ROS suppression reduced these changes, suggesting that oxidative stress acts upstream of the autophagic response. However, the study did not establish whether autophagy itself was protective or injurious.

Yuan et al. subsequently showed that aldosterone-induced ER stress and CHOP-dependent apoptosis occurred alongside increased autophagy-associated activity and that pharmacologic inhibition of autophagy aggravated podocyte apoptosis (A1/C1/G0/E1/M1) [90]. More direct evidence was provided by Bai et al., who used tandem mRFP-GFP-LC3 imaging together with *Atg5* knockdown and pharmacologic manipulation to demonstrate increased autophagic flux and a protective effect against nephrin loss and apoptosis in aldosterone-treated podocytes (A2/C1/G1/E2/M1) [91].

Wang et al. extended this mechanism to mineralocorticoid-induced hypertension [79]. Chloroquine-supported LC3 analysis demonstrated enhanced autophagic flux, while FOXO1–RAB7 signaling and P300-dependent FOXO1 acetylation promoted interactions with ATG7. Disruption of *Atg7* or pharmacologic inhibition of autophagy aggravated podocyte injury, whereas preservation of autophagy was protective. The inclusion of an aldosterone/salt hypertensive model increases its disease relevance (A2/C1/G1/E2/M2), although in vivo autophagy measurements were largely performed at the glomerular rather than podocyte-specific level [79].

Thus, the aldosterone literature provides comparatively consistent evidence that ROS- and FOXO1-associated autophagic flux functions as an adaptive response to mineralocorticoid stress. Importantly, this contrasts with the impaired flux observed during chronic Ang II-induced hypertension. These findings argue against describing podocyte autophagy in HTN-CKD as uniformly activated or suppressed. Rather, the direction of flux appears to depend on the nature and duration of the hypertensive stimulus, while preservation of effective autophagic quality control is protective in both settings.

### 6.4. Mechanical Stress

Mechanical stress provides a third potential route through which hypertension may alter podocyte autophagy. In cultured human podocytes, prolonged stretch reduced cellular adhesion, integrin β1, LC3 puncta, LC3-II, and ATG5 while activating PI3K/AKT/mTOR signaling; MR antagonism or PI3K/mTOR inhibition partially restored autophagy-associated markers and adhesion [40]. This study supports a link between mechanical loading, MR–mTOR signaling, and podocyte autophagy-associated machinery (A1/C1/G0/E1/M1) [40].

However, dynamic flux was not measured, and the findings have not been reproduced in an in vivo hypertensive model. Mechanical stress should therefore be considered a biologically plausible contributor to altered podocyte autophagy during glomerular hypertension rather than an established HTN-CKD mechanism.

### 6.5. Podocyte Mitophagy: An Evidence Gap

Mitochondrial injury is an important component of podocyte stress, creating a potential requirement for selective mitochondrial quality control. In palmitic acid-treated podocytes, PINK1/PRKN-associated mitophagy was linked to reduced mitochondrial ROS and apoptosis [92]. Similarly, FOXO1-associated PINK1/PRKN signaling supported mitochondrial quality control in diabetic mice and high-glucose-treated podocytes [93]. These studies are useful mechanistic comparators but provide no direct evidence for hypertensive kidney disease (both A1/C1/G0/E0/M0) [92,93]. They should therefore not be used as evidence that podocyte mitophagy is impaired in HTN-CKD. At present, direct demonstration of altered podocyte mitophagic flux in a hypertensive model appears to be lacking. Moreover, evidence that basal renal mitophagy can occur independently of PINK1 cautions against assuming that the PINK1/PRKN pathway necessarily represents the dominant mitophagy mechanism in hypertensive podocytes [25].

Accordingly, defective podocyte mitophagy in HTN-CKD remains a plausible but unproven hypothesis. Future studies using podocyte-resolved mitophagy reporters together with hypertensive models and genetic manipulation will be required to determine whether mitochondrial clearance is altered and whether such alterations causally contribute to hypertensive podocytopathy. The podocyte studies discussed in Section 6.1, Section 6.2, Section 6.3, Section 6.4 and Section 6.5 and their ACGEM grades are summarized in Table 3.

**Table 3:** Evidence for podocyte autophagy in hypertensive kidney disease. Studies are organized by the predominant hypertensive or hypertension-relevant stress axis and graded using the multidimensional ACGEM framework. A (Autophagy/mitophagy measurement) reflects the strength of autophagy assessment, ranging from static markers or morphology (A1) to dynamic flux measurements using lysosomal blockade or tandem fluorescent reporters (A2). C (Cell-type resolution) indicates whether findings are localized to podocytes, with higher scores assigned to podocyte-specific in vivo approaches. G (Genetic manipulation) reflects direct genetic perturbation of autophagy-related machinery, such as *Atg5* or *Atg7*. E (Experimental causality) evaluates whether modulation of autophagy alters podocyte injury or survival rather than merely correlating with it. M (Model relevance) denotes relevance to hypertensive kidney disease, with M0 indicating a nonhypertensive comparator model, M1 a hypertension-relevant in vitro or indirect model, and M2 an in vivo hypertensive or hypertension-associated renal injury model. Scores are interpreted independently across dimensions rather than combined into a single hierarchy. Ang II, angiotensin II; MR, mineralocorticoid receptor; SHR, spontaneously hypertensive rat; ROS, reactive oxygen species; TEM, transmission electron microscopy; QYYYG, Qian Yang Yu Yin granule.

Stress Axis	Study	System	Readout	ACGEM	Interpretation	Key Caveat
Basal homeostasis	Hartleben et al. [14]	Podocyte-specific *Atg5* KO mice	Aggregates, ER stress, glomerulosclerosis	A2/C2/G2/E2/M0	Basal autophagy is required for podocyte maintenance	Not a hypertensive model
Ang II, early	Yadav et al. [81]	Cultured podocytes plus Ang II	LC3, autophagosomes, ROS	A1/C1/G0/E0/M1	Ang II can induce stress-associated autophagy	In vitro; no flux; no chronic hypertension
Ang II, early	Seong et al. [85]	Mouse podocytes plus Ang II	LC3A/B-II, BECN1, ATG5 siRNA, apoptosis	A1/C1/G1/E2/M1	Autophagy precedes and restrains apoptosis	Short-term in vitro
Ang II, early/therapeutic	Ma et al., 2023 (QYYYG) [86]	SHR plus Ang II-treated rat podocytes; TRPC6 overexpression	GFP-LC3 puncta, LC3-II/I, BECN1, p62; TRPC6–CaMKKβ–AMPK–mTOR	A1/C1/G0/E1/M2	QYYYG reduces Ang II-associated autophagy markers and podocyte injury through TRPC6–CaMKKβ–AMPK–mTOR-associated signaling	No dynamic flux; TRPC6 manipulation establishes upstream signaling but not autophagy-dependent protection
Ang II, early	Gao et al., 2017 [84]	Cultured podocytes plus Ang II	ROS, TRPC6-mediated Ca^2+^ influx, LC3-II/I, BECN1, p62; rapamycin/3-MA	A1/C1/G0/E1/M1	ROS–TRPC6–Ca^2+^ signaling induces an autophagy-associated response; pharmacologic enhancement improves cell viability	No dynamic flux; pharmacologic causality only
Ang II, chronic	Bensaada et al. [78]	Chronic Ang II mice; podocyte *Atg5* KO; calpastatin	Autophagic flux, calpain/calpastatin	A2/C2/G2/E2/M2	Chronic Ang II impairs glomerular flux; podocyte autophagy is protective	Ang II-dominant; not generalizable to MR/salt hypertension
Aldosterone/MR	Wang et al., 2016 [79]	Aldosterone-treated podocytes; in vivo injury model	LC3-II, flux with lysosomal inhibition, FOXO1, RAB7, ATG7	A2/C1/G1/E2/M2	Aldosterone enhances flux as a compensatory protective response	Aldosterone-injury model, not chronic HTN-CKD
Aldosterone/MR	Bai et al., 2016 [91]	MPC5 podocytes plus aldosterone	Tandem mRFP-GFP-LC3, TEM, LC3/p62; 3-MA and Atg5 siRNA; nephrin/apoptosis	A2/C1/G1/E2/M1	Early ROS-induced autophagic flux is an adaptive response that delays aldosterone-induced podocyte injury	Cell-culture model only; no chronic hypertension
Aldosterone/MR	Yuan et al. [90]	Aldosterone/MR podocyte injury	ROS, ER stress, CHOP, autophagy markers	A1/C1/G0/E1/M1	Autophagy-associated activity is cytoprotective	Flux not assessed
Mechanical stress	Li et al. [40]	Human podocytes plus stretch, with or without spironolactone	LC3 puncta, ATG5, MR, PI3K/AKT/mTOR, adhesion	A1/C1/G0/E1/M1	Stretch-MR-mTOR signaling reduces autophagy markers and adhesion	In vitro; designed in a diabetic context; no in vivo validation
Mitophagy	Jiang et al. [92]	Palmitic acid-treated podocytes	PINK1/PRKN, mitochondrial ROS, apoptosis	A1/C1/G0/E0/M0	Mitophagy limits lipotoxic podocyte injury	Model-relevance flag: lipotoxic, not hypertensive
Mitophagy	Li et al. [93]	Streptozotocin diabetic mice; high-glucose podocytes	FOXO1, PINK1, PRKN, mitochondrial morphology	A1/C1/G0/E0/M0	FOXO1 supports podocyte mitophagy	Model-relevance flag: diabetic, not hypertensive

Abbreviations: Ang II, angiotensin II; CQ, chloroquine; ER, endoplasmic reticulum; MR, mineralocorticoid receptor; SHR, spontaneously hypertensive rat. ACGEM: A, autophagy/mitophagy assessment; C, cell-type resolution; G, genetic manipulation; E, evidence for functional causality; M, relevance of the experimental model to hypertensive kidney disease. Scores range from 0 to 2 where applicable, with higher values indicating stronger evidence within each domain.

## 7. Autophagy and Mitophagy in Proximal Tubular Epithelial Cells

PTECs depend on mitochondrial oxidative metabolism to sustain solute reabsorption, so preservation of mitochondrial homeostasis is central to tubular integrity. In hypertensive kidney disease, tubular mitochondrial stress arises from converging insults [94]: persistent RAAS activation promoting oxidative and mitochondrial injury [95]; increased filtered protein burden after glomerular barrier damage [55]; and progressive microvascular rarefaction with fibrotic thickening of the interstitium, which limits oxygen delivery [35,96].

Autophagy and mitophagy may therefore initially function as adaptive quality-control mechanisms, while chronic stress may eventually challenge their degradative capacity. However, application of the ACGEM framework reveals a substantial gap between this biologically plausible model and the strength of the available experimental evidence.

### 7.1. Context-Dependent Autophagy Responses to Hypertensive Stress

Several mechanistic studies support an adaptive role of tubular autophagy during acute Ang II exposure. Sugawara et al. showed that nonpressor Ang II activated AT1R–ULK1-associated autophagy in proximal tubules and increased tolerance to subsequent ischemia–reperfusion injury (A1/C1/G0/E1/M1) [97]. Peng et al. further demonstrated that ATG5-dependent autophagy restrained Ang II-induced NF-κB activation and inflammatory cytokine production in tubular epithelial cells (A1/C1/G1/E2/M1) [98]. Similarly, PHB2 manipulation indicated that mitophagy can limit mitochondrial dysfunction and NLRP3 inflammasome activation during Ang II stress (A1/C1/G1/E2/M1) [99]. Together, these studies provide mechanistic support for a protective tubular response, but their model relevance remains limited because the principal mechanistic experiments were cellular or were not performed in established hypertensive kidney injury.

Whole-kidney studies in chronic Ang II models have reported heterogeneous autophagy-associated changes, as discussed in Section 5.1 [63,64,65]. Because these studies relied on static, non-cell-type-resolved measurements, they cannot establish the direction of autophagic flux specifically in proximal tubular cells.

More direct evidence for altered flux comes from Li et al., who studied SHRs subjected to exercise together with Ang II-treated HK-2 cells. Moderate-intensity exercise improved renal dysfunction and fibrosis while reducing HIF-1α-associated autophagy, and HIF-1α manipulation and chloroquine-based experiments supported a mechanistic relationship between HIF-1α and autophagic activity (A2/C1/G0/E1/M2) [37]. Importantly, however, dynamic flux interrogation was performed mainly in HK-2 cells rather than directly in hypertensive kidneys. Thus, this study provides relatively strong evidence that prolonged Ang II signaling can produce excessive or maladaptive autophagic activity in tubular cells, but does not establish the state of tubular flux in vivo.

Cai et al. provide a complementary clue. In SHR kidneys and Ang II-treated NRK-52E cells, changes in LC3-II, BECN1, and SQSTM1/p62 accompanied oxidative stress and fibrosis, while chloroquine and rapamycin were used to pharmacologically interrogate autophagy. *Cordyceps cicadae* attenuated these abnormalities together with renal injury (A1/C1/G0/E1/M2) [34]. The concomitant accumulation of LC3-II and p62 is compatible with inefficient autophagic processing and therefore raises the possibility of a clearance-side defect during chronic hypertensive stress. However, because degradative flux was not directly quantified using a validated dynamic assay, these findings demonstrate autophagy-associated dysregulation rather than definitive impairment of autophagic clearance.

Takakura et al. extended these observations to salt-sensitive hypertension [72]. In Dahl salt-sensitive rats, renal injury was accompanied by changes in LC3-II/LC3-I, SQSTM1/p62, tubular LC3 staining, cathepsin D, and mitochondrial proteins, while *Cordyceps militaris* modified these abnormalities together with renal injury (A1/C1/G0/E1/M2) [72]. The study is notable for combining a highly disease-relevant salt-sensitive hypertensive model with tubular localization and assessment of a lysosomal protease. However, the measurements remained static, and neither autophagic flux nor lysosomal degradative capacity was directly tested. Thus, the findings support tubular autophagy–lysosome-associated remodeling during salt-sensitive hypertensive injury but cannot determine whether degradative clearance is enhanced or impaired.

Other intervention studies require similar caution. Protopanaxatriol altered LC3, BECN1, and p62 together with improvement in Ang II-induced renal injury (A1/C1/G0/E1/M2), but without autophagy-specific perturbation or dynamic flux assessment [100]. Nonlethal sonodynamic therapy reduced hypertensive renal fibrosis and tubular epithelial–mesenchymal transition while modifying ROS–PI3K/AKT/mTORC1-associated autophagy (A1/C1/G0/E1/M2), but its causal evidence depends on pharmacological modulators and lacks rigorous dynamic flux measurement [101]. Collectively, these studies show that autophagy-associated pathways track with renal injury and therapeutic responses, but do not by themselves establish whether autophagy is the mediator of protection.

Mineralocorticoid models provide stronger evidence that productive autophagic flux can be protective. In aldosterone-treated PTECs and uninephrectomized aldosterone-treated rats, Cao et al. used lysosomal inhibition together with LC3/p62 and ultrastructural analyses to demonstrate productive autophagy that limited NOX4-dependent oxidative stress and tubular senescence (A2/C1/G0/E1/M1) [102]. Li et al. further demonstrated effective epithelial autophagic flux in aldosterone-treated HK-2 cells and DOCA-salt mice and linked increased flux to reduced tubular EMT and fibrosis (A2/C1/G0/E1/M2) [31]. These studies provide some of the strongest direct flux evidence in hypertension-relevant tubular models, although autophagy causality remains primarily pharmacological rather than genetically established.

Nephron-segment specificity should also be considered when interpreting mineralocorticoid-associated autophagy. Cheema et al. showed that short-term aldosterone administration caused marked accumulation of proteasome-containing protein aggregates predominantly in distal convoluted and connecting tubules, with little corresponding evidence of aggresome or autophagosome formation (A1/C1/G0/E0/M1) [103]. Notably, the aggregates were not observed in proximal tubules, and their formation was prevented by spironolactone. Although this study did not measure dynamic autophagic flux and was not an established hypertensive CKD model, it demonstrates that the capacity to engage protein-clearance pathways in response to mineralocorticoid stress may differ substantially across nephron segments.

Taken together, the tubular literature does not support a simple classification of autophagy as uniformly “activated” or “suppressed” in hypertensive kidney disease. Rather, autophagic responses appear to vary with the nature and duration of hypertensive stress, the nephron segment examined, and—critically—the method used to assess autophagy. The segment-specific response to aldosterone observed by Cheema et al. further cautions against extrapolating findings from one tubular compartment to another. Moreover, static measurements of LC3, BECN1, ATG proteins, or p62 can identify autophagy-associated remodeling but cannot determine whether degradative flux is increased, impaired, or maintained. Thus, both cell-type resolution and dynamic flux assessment are essential for defining the functional state of tubular autophagy in hypertensive kidney disease.

### 7.2. Mitophagy and Mitochondrial Quality Control

Mitophagy may be particularly important in PTECs because their high mitochondrial density makes them vulnerable to accumulation of dysfunctional mitochondria during sustained hypertensive stress. Ang II promotes mitochondrial fragmentation, membrane depolarization, and ROS production, creating a persistent requirement for selective mitochondrial removal.

Recent conceptual advances further emphasize that renal autophagy should not be considered solely as a bulk degradative process. Selective autophagy encompasses cargo-specific pathways including mitophagy, lysophagy, ER-phagy, lipophagy, aggrephagy, and ferritinophagy, whose contributions may differ according to cell type, stress, and disease stage [104]. In hypertensive PTECs, however, mitophagy remains the best studied of these pathways, whereas other forms of selective autophagy are largely unexplored.

Several studies implicate PINK1/PARKIN-associated pathways, although most do not directly measure mitophagic flux. In Ang II-treated tubular cells, SIRT1-associated PINK1/PARKIN signaling reduced mitochondrial dysfunction and cellular senescence (A1/C1/G0/E1/M1), but the corresponding in vivo validation used unilateral ureteral obstruction rather than hypertension [28]. Similarly, reduced PINK1/PARKIN-associated markers and abnormal mitochondrial morphology were observed in SHR nephropathy (A1/C0/G0/E1/M2) [26]. These observations are consistent with altered mitochondrial quality control but cannot determine the direction or efficiency of mitophagic degradation.

Wu et al. provide the strongest evidence for a temporal change in the mitophagy response to Ang II. Mitophagy-associated responses increased during early Ang II exposure but declined during prolonged stimulation, while VEGFR3/HSPA1L signaling restored PARKIN-associated mitochondrial clearance (A1/C1/G0/E1/M2) [27]. The combination of a hypertension-relevant model, genetic manipulation, and experimental perturbation provides substantial mechanistic support for the proposed transition from early adaptation toward later mitophagy dysfunction. Nevertheless, the autophagy measurement itself remains A1 because a dedicated dynamic mitophagy-flux reporter was not used. Thus, the “early adaptation–late failure” model is biologically compelling but remains incompletely demonstrated at the level of mitophagic flux.

Mechanistically strong nonhypertensive studies support the plausibility of this model. Forte et al. used bafilomycin-based flux assessment, tandem mRFP-GFP-LC3 imaging, and UCP2 manipulation to show that effective autophagic flux limits ROS-mediated tubular injury during high-salt exposure (A2/C1/G0/E2/M1) [105]. Likewise, D5-receptor-associated autophagy reduced mitochondrial ROS in renal tubular cells (A2/C1/G0/E1/M0) [106]. These experiments establish mechanisms by which tubular autophagy can preserve mitochondrial homeostasis, but they should be treated as mechanistic comparators rather than direct evidence for hypertensive nephropathy because hypertension and hypertensive renal injury were not established in the experimental systems.

Failure of mitochondrial quality control may contribute to inflammatory and ferroptotic injury in hypertensive kidneys. In human hypertensive nephropathy and experimental Ang II and 2K1C models, tubular mitochondrial damage and cytosolic mtDNA accumulation were linked to STING activation, while epithelial-specific STING deletion attenuated inflammation, fibrosis, and ACSL4-associated ferroptosis [56]. Although this does not establish defective mitophagy as the upstream cause of mtDNA release, mechanistic studies in other tubular injury models support such a possibility. In cisplatin nephrotoxicity, genetic loss of BNIP3-, PINK1-, or PARK2-dependent mitophagy aggravated mitochondrial ROS accumulation, lipid peroxidation, and tubular ferroptosis through the ROS/HO-1/GPX4 axis [107]. Thus, defective mitochondrial clearance is a plausible upstream contributor to ferroptotic injury, but this relationship remains unproven in hypertensive nephropathy.

Human transcriptomic and single-cell analyses similarly identify altered expression of mitophagy-related genes, including PINK1, ULK1, MFN2, SQSTM1, ATG5, and ATG12, particularly in proximal tubular populations [108]. Increased urinary mtDNA in essential and renovascular hypertension further supports mitochondrial injury as a clinically detectable feature [57]. Neither observation, however, measures mitophagic flux. Human evidence therefore supports mitochondrial stress and altered quality-control signaling in hypertensive kidney disease but not yet mitophagic failure itself.

An additional limitation is the field’s reliance on PINK1 and PARKIN abundance as surrogates for mitophagy. Basal mitophagy in metabolically active tissues, including the kidney, can proceed substantially independently of PINK1 [24,25]. Changes in PINK1/PARKIN expression should therefore not be equated with corresponding changes in mitophagic flux.

### 7.3. What the ACGEM Framework Reveals: The Missing Clearance Step

When the tubular literature is evaluated across the five independent ACGEM dimensions, a consistent evidence gap becomes apparent. Disease-model relevance is relatively strong: multiple Ang II, SHR, DOCA-salt, and other hypertension-relevant studies achieve M2. However, high model relevance is rarely combined with direct dynamic flux assessment (A2), tubular resolution (C2), genetic manipulation (G1–2), and autophagy-specific experimental perturbation (E1–2) within the same experiment.

This distinction is critical. High model relevance cannot compensate for limited autophagy measurement. For example, Shen et al., Majumder et al., and several therapeutic studies establish that autophagy-associated proteins change during hypertensive renal injury, but their A1 measurements cannot determine whether degradation is accelerated or blocked [63,64,65,101]. Conversely, Forte et al. provided rigorous dynamic and causal evidence (A2/C1/G0/E2/M1) using high-salt-exposed proximal tubular cells, but the model remained an indirect hypertension-relevant system rather than hypertensive kidney disease in vivo [105]. Even among the strongest M2 studies, important dimensions remain incomplete: Li et al. (2025) achieved A2/C1/G0/E1/M2, but dynamic flux was interrogated mainly in cultured HK-2 cells [37]; Li et al. (2021) achieved A2/C1/G0/E1/M2 in a DOCA-salt setting but without genetic autophagy manipulation [31]; and Wu et al. achieved A1/C1/G0/E1/M2, providing disease-relevant mechanistic evidence but without direct dynamic mitophagy-flux measurement or genetic manipulation of the mitophagy machinery itself [27].

The contrast is particularly evident in Matsui et al., a mechanistically strong but nonhypertensive comparator (A2/C2/G2/E2/M0) [75]. Using inducible PTEC-specific genetic models, in vivo chloroquine-based flux assessment, GFP-LC3, and measures of lysosomal burden and autophagic reserve, this study demonstrated that sustained cargo load can exceed tubular autophagic and lysosomal capacity [75]. The study does not establish that the same phenomenon occurs in hypertension, but it provides an experimental framework for asking whether chronic hypertensive stress similarly overwhelms degradative capacity.

This comparison exposes an important blind spot in the hypertensive literature: the terminal degradative compartment has received substantially less attention than autophagosome formation and upstream signaling. Most studies measure LC3, BECN1, ATG proteins, p62, PINK1/PARKIN, AMPK, or mTOR, whereas direct measurements of lysosomal pH, cathepsin activity, lysosomal morphology, TFEB localization, autophagosome–lysosome fusion, and degradative reserve are largely absent from hypertension-specific models. Consequently, accumulation of LC3-positive structures, p62, or damaged mitochondria cannot determine whether the primary abnormality lies in increased autophagic demand, impaired autophagosome formation, defective lysosomal clearance, or some combination of these processes.

A clearance-limited autophagy model may therefore provide a useful framework for reconciling apparently contradictory observations. During acute or moderate stress, increased autophagosome formation accompanied by sufficient lysosomal capacity could generate productive flux and preserve tubular homeostasis. With persistent Ang II signaling, oxidative stress, mitochondrial injury, protein load, and hypoxia, however, degradative demand may progressively approach or exceed lysosomal capacity. Under such conditions, autophagy-associated signaling and autophagosome formation could remain active while effective cargo clearance becomes insufficient. This scenario could produce the seemingly paradoxical coexistence of increased LC3, p62 accumulation, damaged mitochondria, inflammation, senescence, and fibrosis reported across chronic models.

Importantly, the available ACGEM evidence does not establish this proposed autophagy stagnation as a demonstrated mechanism of hypertensive nephropathy. Rather, it identifies a specific and testable gap in the current literature. Definitive evaluation will require hypertensive models that combine dynamic autophagic and mitophagic flux measurements (A2), tubular cell-type resolution (C2), genetic manipulation of autophagy or lysosomal machinery (G1–2), direct experimental perturbation (E1–2), and high disease-model relevance (M2). Tandem LC3 or mitochondrial flux reporters combined with PTEC-specific genetic models and direct measurements of lysosomal pH, cathepsin activity, TFEB signaling, autophagosome–lysosome fusion, and degradative reserve will be necessary to determine whether progressive hypertensive tubular injury reflects impaired autophagy initiation, insufficient lysosomal clearance, or both. The tubular studies discussed in Section 7.1, Section 7.2 and Section 7.3, together with their ACGEM grades and principal limitations, are summarized in Table 4.

**Table 4:** Autophagy and mitophagy in renal tubular cells under hypertensive stress: evidence characteristics and limitations. Evidence is evaluated across five independent ACGEM domains: autophagy/mitophagy measurement (A), cell-type resolution (C), genetic manipulation (G), experimental autophagy/mitophagy perturbation (E), and model relevance (M), as defined in Table 1. The table summarizes the strength and limitations of the available evidence rather than assigning an overall evidence tier or implying established mechanisms.

Stress Axis	Study	System	Readout	ACGEM	Interpretation	Key Caveat
Ang II/adaptive autophagy	Sugawara et al. [97]	Nonpressor Ang II; proximal tubular cells/mouse kidney	AT1R–ULK1 signaling, LC3-associated autophagy, IRI tolerance	A1/C1/G0/E1/M1	Acute Ang II can induce an adaptive tubular autophagy response that increases stress tolerance	Preconditioning design with IRI endpoint rather than hypertensive nephropathy; no dynamic flux
Ang II/inflammation	Peng et al. [98]	Ang II-treated tubular epithelial cells	ATG5, LC3, NF-κB, inflammatory cytokines; Atg5 manipulation	A1/C1/G1/E2/M1	ATG5-dependent autophagy restrains Ang II-induced inflammatory signaling	In vitro only; static autophagy assessment
Ang II/mitophagy–inflammasome	Xu et al. [99]	Ang II-treated tubular epithelial cells	PHB2 KD/OE, LC3, CQ, mitochondrial–lysosomal colocalization, NLRP3	A1/C1/G1/E2/M1	PHB2-associated mitophagy limits mitochondrial dysfunction and NLRP3 activation	No dedicated dynamic mitophagy-flux reporter; no hypertensive animal model
SHR/HIF-1α	Li et al., 2025 [37]	SHR ± MICT/HIIT; Ang II-treated HK-2 cells	ATG5, LC3B-II, p62, HIF-1α, AKT/mTOR, CQ	A2/C1/G0/E1/M2	HIF-1α-associated excessive autophagy is linked to tubular fibrosis; MICT attenuates this response	Dynamic flux assessed mainly in HK-2 cells rather than hypertensive kidney in vivo; HIF-1α rather than autophagy genes manipulated
Ang II/PTPN1	Zhao et al., 2025 [100]	Ang II-infused mice; Ang II-treated tubular cells	LC3, BECN1, p62, PTPN1–AMPK/mTOR	A1/C1/G0/E1/M2	PTPN1-linked signaling accompanies altered tubular autophagy-associated activity and renal injury	No dynamic flux or autophagy-specific perturbation
SHR/oxidative stress	Cai et al. [34]	SHR kidneys; Ang II-treated NRK-52E cells	LC3-II, BECN1, p62, CQ/rapamycin, SIRT1/FOXO3a	A1/C1/G0/E1/M2	LC3-II/p62 accumulation suggests autophagy dysregulation and raises the possibility of inefficient processing during chronic hypertensive stress	Static readouts do not establish impaired clearance; multicomponent Cordyceps cicadae intervention
Salt-sensitive HTN	Takakura et al., 2017 [72]	Dahl salt-sensitive rats ± Cordyceps militaris	LC3-II/I, p62, tubular LC3, cathepsin D, mitochondrial proteins	A1/C1/G0/E1/M2	Salt-sensitive hypertensive injury is accompanied by segment-resolved tubular autophagy- and mitochondrial-associated remodeling	No direct flux or autophagy-specific perturbation; cathepsin D abundance does not establish lysosomal function
Aldosterone/ROS	Wang et al., 2015 [109]	Aldosterone-treated NRK-52E cells ± NAC/Rg1	LC3-II, BECN1, autophagosomes, ROS, AMPK/mTOR	A1/C1/G0/E0/M1	Aldosterone induces a ROS-associated tubular autophagy response	Cell-only model; productive versus impaired flux remains unresolved
Aldosterone/senescence	Cao et al., 2021 [102]	Aldosterone-treated PTECs; UNx + aldosterone rats	LC3/p62 turnover, CQ, TEM, NOX4, ROS, p21	A2/C1/G0/E1/M1	Productive autophagic flux limits oxidative stress and tubular senescence	Hypertensive phenotype not firmly established; causality largely pharmacologic
DOCA-salt/fibrosis	Li et al., 2021 [31]	DOCA-salt mice; aldosterone-treated HK-2 cells	Tandem RFP-GFP-LC3, AMPK–ULK1, CQ, EMT/fibrosis	A2/C1/G0/E1/M2	Effective epithelial autophagic flux is protective against tubular EMT and fibrosis	No genetic manipulation of autophagy machinery
Ang II/NL-SDT	Liu et al., 2025 [101]	Ang II hypertensive mice; TGF-β1-treated NRK-52E cells	LC3B, puncta, TEM, PI3K/AKT/mTORC1; CQ, 3-MA, rapamycin	A1/C1/G0/E1/M2	Autophagy-associated signaling contributes to the antifibrotic response to NL-SDT	No rigorous reporter-based flux; pharmacologic interventions are nonspecific; in vitro stressor is TGF-β1
Ang II/mitophagy–senescence	Liu et al., 2020 [28]	Ang II-treated NRK-52E cells; UUO rats	PINK1/PARKIN, LC3/p62, mitophagy dye, EX527/Mdivi-1	A1/C1/G0/E1/M1	SIRT1–PINK1/PARKIN-associated mitochondrial quality control limits tubular senescence	Hypertension-relevant evidence mainly in vitro; in vivo validation uses UUO
Prolonged Ang II/mitophagy	Wu et al., 2025 [27]	Ang II mice; Ang II-treated HK-2 cells; HSPA1L manipulation	Mitochondrial PARKIN, LC3/p62, TEM, colocalization	A1/C1/G0/E1/M2	Mitophagy-associated responses appear adaptive early but become impaired with prolonged Ang II exposure; VEGFR3/HSPA1L restores the pathway	No dedicated dynamic mitophagy-flux reporter; temporal failure model remains incompletely demonstrated
Aldosterone/segment specificity	Cheema et al., 2014 [103]	Aldosterone-treated rats ± spironolactone	Distal tubular protein aggregates, LC3, aggresome markers, proteasome 20S, cathepsin D	A1/C1/G0/E0/M1	Aldosterone-induced protein-clearance responses differ markedly across nephron segments, with impaired aggregate handling particularly in DCT/CNT	Distal-tubule comparator rather than PTEC study; short-term aldosterone exposure; no dynamic flux

## 8. Other Compartments: Endothelium, Vasculature, Mesangium, and Immune Cells

The concentration of research effort on podocytes and PTECs is understandable but has left an important gap. The defining histologic lesion of hypertensive nephrosclerosis is vascular, yet the vascular compartment has been the least studied.

### 8.1. Glomerular and Peritubular Endothelium

Endothelial injury is a central component of hypertensive kidney disease, affecting both the glomerular microcirculation and the peritubular capillary network. Sustained pressure, Ang II signaling, oxidative stress, and reduced nitric oxide bioavailability promote endothelial dysfunction, whereas progressive microvascular rarefaction contributes to tissue hypoxia and tubulointerstitial fibrosis [110]. Despite this pathophysiologic importance, the role of endothelial autophagy in hypertensive kidney injury remains poorly defined.

Experimental studies in systemic vascular beds suggest that endothelial autophagy supports vascular homeostasis by preserving eNOS-dependent nitric oxide signaling and limiting oxidative and inflammatory stress [111,112]. Importantly, renal evidence from a nonhypertensive disease model provides stronger cell-specific support for a protective role of endothelial autophagy. Lenoir et al. demonstrated that endothelial-specific deletion of Atg5 aggravated glomerular endothelial injury and capillary rarefaction in diabetic mice and was accompanied by worsening podocyte ultrastructural abnormalities and glomerular injury [80]. Together with complementary podocyte-specific Atg5 experiments, these findings indicate that autophagy in endothelial cells and podocytes contributes cooperatively to maintenance of the glomerular filtration barrier [80]. However, because these experiments were performed in diabetic nephropathy rather than hypertension, they establish the importance of endothelial autophagy in glomerular homeostasis but cannot be considered direct evidence for hypertensive nephrosclerosis.

Accordingly, it remains unknown whether autophagy protects renal endothelial cells during hypertension, becomes maladaptive under prolonged hemodynamic or Ang II stress, or ultimately fails because degradative demand exceeds lysosomal capacity. This question cannot be resolved from whole-kidney LC3 or p62 measurements or from systemic vascular studies alone. Future studies should combine established hypertensive models with endothelial-specific genetic manipulation of autophagy-related genes and dynamic autophagy or mitophagy reporters, while distinguishing glomerular from peritubular endothelial populations. Such approaches will be necessary to determine whether altered endothelial autophagy contributes directly to glomerular capillary injury, microvascular rarefaction, tissue hypoxia, and progression of hypertensive nephrosclerosis.

### 8.2. Vascular Smooth Muscle Cells and Renal Arteriolar Remodeling

Vascular smooth muscle cells (VSMCs) warrant particular consideration in hypertensive kidney disease because arteriolar wall thickening, hyaline change, luminal narrowing, and impaired autoregulation are characteristic components of hypertensive nephrosclerosis [113,114]. Within the renal resistance vasculature, especially interlobular arteries and preglomerular and afferent arterioles, VSMCs regulate vascular tone and adapt structurally to sustained increases in pressure and neurohumoral stimulation [114]. Chronic hypertensive stress can drive VSMCs away from a quiescent contractile phenotype toward synthetic, proliferative, senescent, or osteogenic states [115]. Because autophagy participates in each of these phenotypic transitions, altered VSMC autophagy represents a plausible link between systemic hypertension and progressive renal arteriolosclerosis [116].

Experimental evidence from systemic vascular models indicates that the effect of autophagy depends strongly on the initiating stress and VSMC state. Ang II induces autophagy through an AT1R–RhoA/ROCK-dependent pathway, and pharmacological inhibition of autophagy attenuates Ang II-induced VSMC hypertrophy [117]. Mechanical stress provides a second hypertension-relevant stimulus. In abdominal aortic coarctation-induced hypertensive rats and primary VSMCs exposed to pathological cyclic stretch, Li et al. demonstrated increased autophagic flux together with suppression of mTOR signaling following loss of the nuclear-envelope proteins lamin A/C and emerin. Bafilomycin A1 or chloroquine reduced VSMC proliferation, supporting a role for mechanically induced autophagy in proliferative vascular remodeling (A2/C1/G0/E1/M1) [118]. More recently, SOX6-mediated autophagy was shown to promote transition from a contractile to a synthetic VSMC phenotype, thereby enhancing vascular remodeling and contributing to elevated blood pressure [119]. Collectively, these findings suggest that stress-induced autophagy can facilitate hypertrophic, proliferative, and phenotypic remodeling under conditions relevant to hypertension.

However, increased autophagy should not be interpreted as uniformly pathogenic in VSMCs. VSMC-specific deletion of *Atg7* accelerates senescence, neointima formation, and atherogenesis [120] and aggravates Ang II-associated arterial remodeling [121], indicating that constitutive autophagy is required to preserve VSMC homeostasis. Autophagy can also restrain osteogenic phenotypic switching. In H_2_O_2_-stressed VSMCs and rats with vascular calcification, luteolin increased BECN1 and LC3-II/LC3-I and suppressed oxidative stress and osteogenic transformation through SIRT1/CXCR4-associated signaling; chloroquine partially attenuated these effects (A1/C1/G0/E1/M0) [122]. Thus, the relationship between autophagy and VSMC phenotype is likely biphasic and state dependent: basal autophagic quality control may prevent senescence and maladaptive differentiation, whereas stress-induced autophagy may, in some settings, support hypertrophy, proliferation, or acquisition of a synthetic phenotype.

This distinction may be particularly relevant to the renal microvasculature. Phenotypic conversion of contractile VSMCs toward synthetic or senescent states could contribute to medial thickening, extracellular-matrix deposition, reduced arteriolar compliance, and progressive luminal narrowing. In preglomerular vessels, such remodeling could further impair renal autoregulation and alter transmission of systemic pressure to the glomerular circulation, whereas progressive vascular narrowing may exacerbate downstream ischemia, capillary rarefaction, tubular hypoxia, and fibrosis. Autophagy may therefore influence hypertensive kidney disease not only through parenchymal cell survival but also by determining how renal vascular smooth muscle responds to chronic mechanical and neurohumoral stress.

Importantly, this proposed role remains largely extrapolative. Most mechanistic studies of VSMC autophagy have used aortic tissue or cultured systemic VSMCs rather than VSMCs isolated from renal resistance vessels, and direct measurements of autophagic flux in afferent or other intrarenal arteriolar VSMCs during hypertension are essentially lacking. Future studies should therefore combine established hypertensive models with renal vascular cell-type resolution—for example, VSMC-specific manipulation of Atg5 or Atg7, lineage tracing of contractile-to-synthetic phenotypic switching, and tandem LC3 or mitochondrial flux reporters—and relate these measurements directly to arteriolar wall thickening, hyalinosis, luminal narrowing, and renal autoregulatory dysfunction. Such studies would extend the cell-specific framework of hypertensive kidney disease from glomerular and tubular compartments to the vascular wall and determine whether VSMC autophagy is a driver, compensatory response, or stage-dependent modifier of hypertensive arteriolosclerosis.

### 8.3. Mesangial Cells

Evidence from mesangial cells further illustrates the context-dependent nature of autophagy in hypertensive stress. In human glomerular mesangial cells, Ding et al. found that Ang II increased LC3- and BECN1-associated autophagy markers together with ERK activation and cellular proliferation, whereas calycosin attenuated both responses (A1/C1/G0/E0/M1) [123]. Because autophagy was assessed only by static markers and was not directly perturbed, however, this study does not establish that autophagy mediated the proliferative phenotype.

Stronger mechanistic evidence was provided by Yang et al., who used tandem GFP-RFP-LC3 imaging together with 3-MA and rapamycin to demonstrate dynamic autophagic responses in Ang II-treated human mesangial cells (A2/C1/G0/E1/M1) [124]. In this setting, STAT3/mTOR-associated autophagy promoted cellular senescence, whereas pharmacological inhibition of autophagy attenuated the senescent phenotype [124]. These findings indicate that sustained autophagic activity can become maladaptive in Ang II-stressed mesangial cells, although the evidence remains confined to an in vitro system without hypertensive kidney validation.

By contrast, Fan et al. identified a protective form of cargo-selective autophagy in Ang II-induced renal injury [125]. Carnosol promoted p62-dependent autophagic degradation of KEAP1, thereby activating NRF2/HO-1 antioxidant signaling and limiting oxidative injury (A1/C1/G1/E2/M2) [125]. The use of p62 silencing together with lysosomal and autophagy inhibitors provides relatively strong causal support for this pathway. Importantly, however, the study primarily defines selective p62/KEAP1 cargo handling rather than global autophagic flux, and most of the mechanistic dissection was performed in cultured mesangial cells.

Taken together, these studies argue against assigning a uniformly protective or pathogenic role to mesangial autophagy. Rather, its functional consequences appear to depend on the form of autophagy engaged and the downstream cargo being processed: sustained bulk autophagy may contribute to proliferation or senescence under Ang II stress, whereas selective p62-dependent degradation of KEAP1 can support antioxidant defense. This distinction between bulk and cargo-selective autophagy may be particularly important when interpreting autophagy-associated signaling in the mesangial compartment.

### 8.4. Infiltrating Immune Cells

Autophagy shapes macrophage polarization, inflammasome activation, and T cell function, all of which are implicated in hypertensive renal injury. This creates a specific interpretive difficulty: an autophagy-restoring agent may act principally on infiltrating leukocytes rather than on resident renal cells, and whole-kidney lysates cannot distinguish the two. Reports linking autophagy to NLRP3 inflammasome activity and macrophage polarization in hypertensive kidneys are compatible with either interpretation [126].

### 8.5. Minimum Reporting Standards for Future Work

The gaps above are not a critique of individual studies but a call for structural change in how autophagy is reported in this field. We suggest that future primary studies of autophagy in hypertensive kidney disease should include, at minimum: a flux assay in the relevant cell type, using tandem reporters (mRFP/mCherry-GFP-LC3, mt-Keima, mito-QC) or lysosomal-inhibitor clamps with quantified LC3-II accumulation; at least one lysosomal readout (cathepsin B or D activity, lysosomal pH, LAMP1 morphology, or TFEB nuclear localization), so that formation and clearance can be distinguished; cell-type localization, either through immunofluorescence in tissue or through cell-type-specific genetic manipulation; and explicit model-relevance justification where the model does not reproduce the hypertensive stimulus of interest, for example unilateral ureteric obstruction, ischemia-reperfusion, or diabetes.

## 9. A Working Hypothesis: Could Lysosomal Clearance Be the Rate-Limiting Step?

### 9.1. Autophagic Stagnation as a Clearance-Limited State in Hypertensive Kidney Disease

A central limitation of the hypertensive kidney literature is that most studies assess autophagosome-associated proteins or upstream signaling rather than terminal degradation. LC3-II, BECN1, ATG proteins, and SQSTM1/p62 indicate autophagy-associated remodeling but cannot distinguish altered autophagosome formation from impaired lysosomal clearance [16]. Consistent with the multidimensional ACGEM framework, even studies with high disease-model relevance (M) rarely combine dynamic flux assessment (A2), cell-type resolution (C2), and direct measures of lysosomal function.

This distinction may be particularly relevant in chronic hypertensive injury, where persistent Ang II signaling, oxidative and mitochondrial stress, hypoxia, protein overload, and increased tubular workload continuously increase autophagic demand. The relevant question may therefore be not simply whether autophagy is activated or suppressed, but whether lysosomal degradative capacity remains sufficient for the increasing cargo burden. Early or moderate stress may be accommodated by increased autophagic turnover, whereas persistent injury could produce a demand–capacity mismatch in which LC3-positive structures, SQSTM1/p62, and damaged organelles accumulate despite continued upstream signaling. At more advanced stages, impairment of autophagosome formation may also coexist. Thus, heterogeneous static-marker patterns are compatible with different positions along a dynamic formation–clearance continuum, although they do not themselves demonstrate autophagic stagnation.

Direct pathological evidence for a lysosomal clearance defect in human hypertensive nephrosclerosis is currently absent. In particular, no study has, to our knowledge, directly assessed lysosomal function or cell-type-resolved autophagic clearance in renal biopsy tissue from patients with biopsy-confirmed classical hypertensive nephrosclerosis. Clearance-limited autophagy should therefore be regarded as an extrapolative, testable working hypothesis rather than an established disease mechanism.

### 9.2. Mechanistic Precedents for Clearance-Limited Autophagy

Mechanistic precedent comes primarily from other forms of renal stress. Aging and obesity provide the clearest examples of “autophagic stagnation,” in which sustained autophagic demand is accompanied by SQSTM1/p62 accumulation, defective lysosomal acidification, and retention of undegraded organelles and phospholipids within enlarged lysosomes [127,128,129]. Human CKD tissue from patients with higher BMI similarly shows proximal tubular vacuolation and reduced nuclear TFEB localization, although these findings derive from obesity-associated disease and should not be extrapolated directly to hypertensive nephrosclerosis [129].

Experimental obstructive nephropathy provides complementary causal evidence. Reduced TFEB disrupts ATP6V0C- and STX17/VAMP8-dependent autophagosome–lysosome processing, whereas restoration of TFEB improves autophagic flux and attenuates tubular G2/M arrest and fibrosis [130].

APOL1-associated kidney disease provides an additional mechanistic precedent for lysosomal vulnerability. In cultured human podocytes, expression of APOL1 G1 and G2 risk variants increased lysosomal membrane permeability and promoted podocyte injury [131]. Complementary findings in genotype-defined primary endothelial cells showed contraction of the lysosomal compartment and accumulation of autophagosomes in cells carrying two APOL1 risk alleles [132]. However, APOL1-associated disease is genetically determined and mechanistically distinct from classical hypertension-driven arterionephrosclerosis; these findings therefore provide mechanistic precedent for lysosomal dysfunction rather than direct evidence for hypertensive nephrosclerosis.

CINAC provides a separate pathological precedent. Chronic interstitial nephritis in agricultural communities (CINAC) is a chronic tubulointerstitial kidney disease occurring predominantly in agricultural populations and is not attributable to conventional causes such as hypertension or diabetes. Its etiology remains debated, with occupational heat stress and recurrent dehydration, agrochemical or other toxic exposures, and multifactorial environmental mechanisms having been proposed [133,134]. Importantly, renal biopsy studies have demonstrated enlarged, dysmorphic lysosomes containing electron-dense aggregates in proximal tubular epithelial cells, identifying lysosomal injury as a prominent pathological feature [134]. Because CINAC arises in an epidemiologically and etiologically distinct setting, however, these findings should be regarded as pathological precedent for renal lysosomal vulnerability rather than direct evidence for hypertensive nephrosclerosis.

Proteinuric overload provides another mechanistic precedent relevant to lysosomal demand. Nolin et al. demonstrated that excessive protein exposure can impair proximal tubular autophagic flux and alter lysosomal loading [55]. Although proteinuria may occur in hypertensive CKD, proteinuric overload represents a secondary stress resulting from glomerular barrier injury and does not establish a primary lysosomal defect caused by hypertension.

Taken together, these etiologically distinct conditions do not constitute convergent human evidence for a lysosomal defect in hypertensive nephrosclerosis. Rather, they provide mechanistic or pathological precedents showing that lysosomal processing can become vulnerable under sustained renal stress. Whether a comparable defect occurs in classical hypertensive nephrosclerosis remains unknown and requires direct pathological validation.

### 9.3. A Working Model for Hypertensive CKD

On this basis, we propose that in at least a subset of hypertension-attributed CKD, the rate-limiting step in autophagic quality control may shift from autophagosome formation toward downstream clearance as injury becomes chronic. Persistent oxidative, mitochondrial, proteinuric, and metabolic stress could progressively increase cargo burden relative to lysosomal degradative reserve in podocytes and proximal tubular epithelial cells. Under these conditions, accumulation of LC3-II, SQSTM1/p62, and autophagosomes could reflect impaired degradation rather than simply increased or decreased initiation.

**Figure 2:**
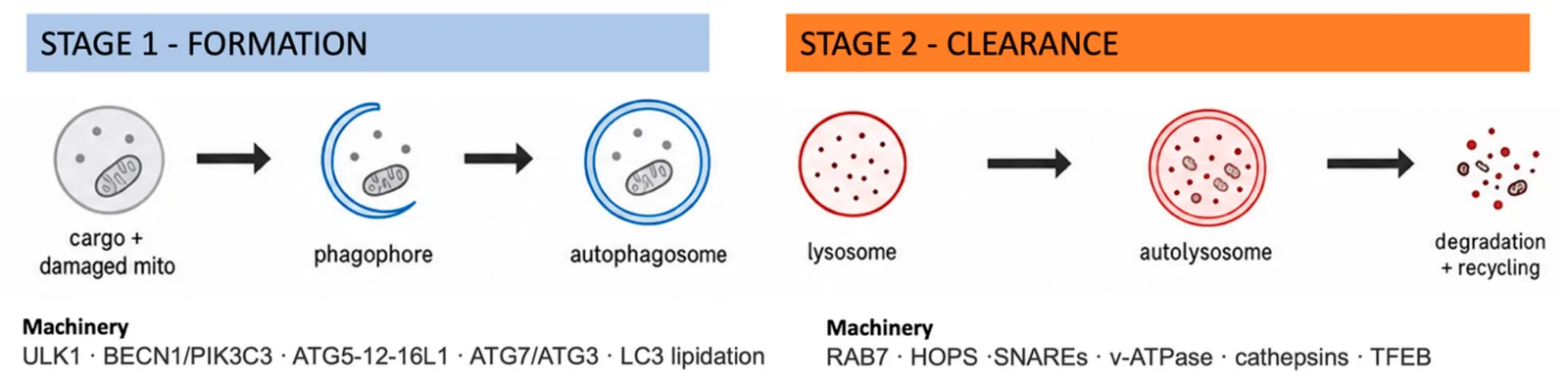
Formation and clearance stages of the autophagy pathway and their relevance to interpretation of the hypertensive kidney literature. Autophagy is shown schematically as two analytically distinguishable but biologically coupled stages. Stage 1 (formation) encompasses initiation, phagophore nucleation and elongation, and formation of the cargo-containing autophagosome. Representative machinery includes ULK1, the BECN1–PIK3C3 complex, the ATG5–ATG12–ATG16L1 and ATG7–ATG3 conjugation systems, and LC3 lipidation. Stage 2 (clearance) comprises autophagosome–lysosome fusion, lysosomal acidification and hydrolysis, cargo degradation, and recycling. Representative machinery includes RAB7, the HOPS tethering complex, SNARE proteins, vacuolar H+-ATPase (v-ATPase), lysosomal cathepsins, and the lysosomal biogenesis regulator TFEB.

This interpretation is compatible with one of the strongest direct flux observations in the hypertensive glomerular literature (A2), in which chronic Ang II impaired glomerular autophagic flux and podocyte autophagy was protective [78]. However, that study did not localize the defect specifically to clearance because impaired autophagosome formation was not excluded. Aldosterone-induced enhancement of podocyte flux through FOXO1 and RAB7 further indicates that autophagosome maturation and lysosomal fusion are actively regulated under mineralocorticoid stress [79]. Together, these findings justify investigation of a clearance-side defect but do not establish one (Figure 2).

This distinction is important because commonly used static readouts, including LC3-II, BECN1, ATG proteins, and SQSTM1/p62, indicate autophagy-associated remodeling but do not by themselves distinguish altered autophagosome formation from impaired downstream clearance. Dynamic flux measurements and direct assessment of lysosomal competence are therefore required to localize the rate-limiting defect. In the hypertension-relevant studies reviewed here, lysosomal function has rarely been assessed directly despite frequent measurement of upstream or autophagosome-associated markers. Observations from APOL1-associated nephropathy, CINAC, proteinuric overload, aging, and obesity provide mechanistic or pathological precedent for lysosomal vulnerability but are etiologically distinct and should not be considered direct evidence for classical hypertensive nephrosclerosis. Accordingly, the proposed clearance-limited model is presented as an extrapolative, falsifiable working hypothesis that remains to be validated in biopsy-confirmed classical hypertensive nephrosclerosis.

### 9.4. The Differential Therapeutic Prediction

The principal value of this hypothesis is that it generates a testable prediction that differs from an initiation-limited model. The distinction is deliberately schematic because formation and clearance are biologically coupled and may both be affected to different degrees; the key question is which step limits net degradative throughput.

**Figure 3:**
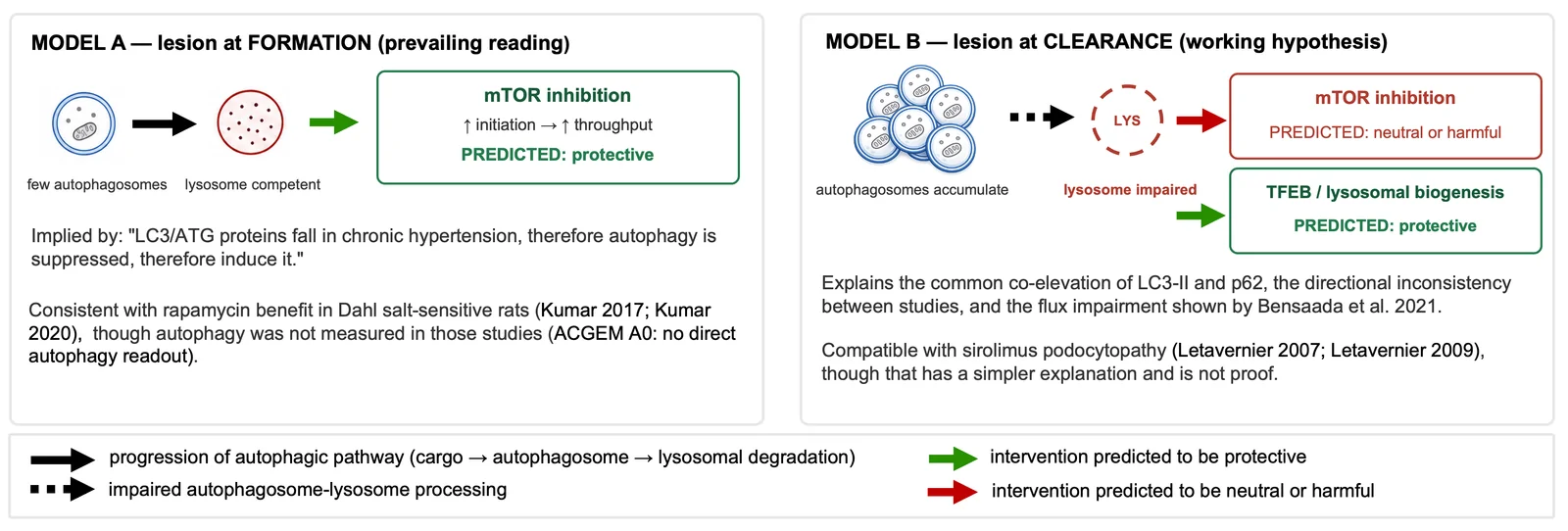
The differential therapeutic prediction. Two alternative models of impaired autophagic throughput generate different predictions for initiation-directed therapy. If autophagosome formation is rate limiting (Model A), enhancing initiation would be expected to increase net autophagic degradation and confer protection; this reading is consistent with the renoprotective effect of rapamycin in Dahl salt-sensitive rats (shown in the figure as Kumar 2017 and Kumar 2020) [29,30], although autophagy was not directly measured in those studies and they were therefore graded A0 for autophagy/mitophagy measurement. If lysosomal clearance is rate limiting (Model B), increasing autophagosome formation without restoring lysosomal capacity could instead increase autophagosome and cargo burden without increasing degradative throughput, making restoration of lysosomal function or biogenesis a more rational therapeutic strategy. Model B is compatible with the impaired glomerular autophagic flux reported under chronic angiotensin II exposure (shown as Bensaada et al. 2021) [78] and with sirolimus-associated podocytopathy (shown as Letavernier 2007 and Letavernier 2009) [135,136], although the latter has a simpler nonautophagic explanation and does not constitute proof. Arrows are used as follows: solid black arrows indicate progression of the autophagic pathway from cargo sequestration through autophagosome formation to lysosomal degradation; the dashed black arrow indicates impaired autophagosome–lysosome processing; the green arrow indicates an intervention predicted to be protective; and the red arrow indicates an intervention predicted to be neutral or harmful. The dashed red outline of the lysosome (LYS) in Model B denotes a functionally impaired degradative compartment, in contrast to the solid outline used in Model A. The diagram is intentionally schematic because autophagosome formation and clearance are biologically coupled and may both be affected to different degrees. The relevant question is therefore which step limits net degradative throughput. Model B represents the working hypothesis developed in Section 9.3, Section 9.4 and Section 9.5 and should not be interpreted as an established mechanism.

If autophagosome formation is limiting, interventions that enhance initiation would be expected to increase net autophagic degradation and confer protection. If clearance is limiting, however, increasing autophagosome formation without restoring lysosomal capacity could increase autophagosome and cargo burden without increasing degradative throughput. The two models therefore predict different responses to the same intervention (Figure 3).

Clinical experience with systemic mTORC1 inhibition is compatible with, but does not establish, this distinction. Sirolimus has been associated with de novo proteinuria and FSGS after kidney transplantation and can disrupt pathways required for podocyte integrity [135,136]. These effects are most readily explained by nonautophagic actions of sirolimus and should not be interpreted as evidence for a clearance defect; they simply caution against assuming that indiscriminate enhancement of autophagy initiation will necessarily benefit podocytes.

If lysosomal clearance proves limiting, TFEB-dependent lysosomal biogenesis, maintenance of lysosomal acidification, and the RAB7- and HOPS-dependent fusion machinery become rational experimental targets [18]. To our knowledge, TFEB activation has not yet been tested directly in a hypertensive kidney model.

### 9.5. How to Test and Falsify the Hypothesis

The hypothesis can be tested using existing experimental approaches. First, chronic Ang II or mineralocorticoid-salt models carrying cell-type-resolved tandem mCherry-GFP-LC3 or mt-Keima reporters could determine whether the autolysosome-to-autophagosome ratio falls while autophagosome formation remains preserved. Such a pattern would support a clearance-side lesion, whereas reduced autophagosome formation with preserved clearance would argue against it.

Second, lysosomal competence should be assessed at matched disease stages using complementary functional readouts, including cathepsin B and D activity, lysosomal pH, LAMP1 morphology, and nuclear TFEB localization. A progressive decline in lysosomal function preceding overt structural injury would be expected under a clearance-limited model.

Third, initiation-directed and clearance-directed interventions should be compared within the same hypertensive model using cell-type-resolved flux, rather than static markers, as the primary mechanistic endpoint. Rapamycin is an imperfect initiation probe in podocytes because basal podocyte autophagy is regulated predominantly through AMPK–ULK1 rather than canonical mTORC1 signaling, and its effects are time-dependent [137,138]. AMPK–ULK1-directed manipulation may therefore provide a more interpretable initiation comparator, whereas mTORC1 inhibition should be evaluated with concurrent AMPK and flux measurements.

The model would be weakened if autophagosome formation, rather than clearance, proves limiting; if lysosomal function remains intact despite static-marker changes; or if selective enhancement of initiation consistently restores flux and renal structure. Formation and clearance are also biologically coupled through lysosomal–TFEB–mTORC1 feedback, so their separation here is an analytical framework for identifying the rate-limiting step rather than a claim that the two processes are mechanistically independent [18,139].

Most importantly, the proposed clearance defect remains an extrapolation until lysosomal abnormalities are demonstrated directly in biopsy-confirmed classical hypertensive nephrosclerosis. Human pathological validation should therefore be considered essential before this framework is extended beyond a mechanistic working hypothesis.

## 10. Therapeutic Implications: Separating Attribution from Association

Autophagy is an attractive therapeutic target, and a substantial number of agents have been reported to protect the hypertensive kidney through autophagy. The analysis above imposes several constraints on how such claims should be read.

### 10.1. The Blood Pressure Confounder

Several interventions reported to restore renal autophagy also lower blood pressure, including spironolactone [126] and VEGFC in the VEGFR3 study [27]. Where blood pressure falls, renoprotection has an obvious alternative explanation, and none of these studies included a pressure-matched control arm. Blood pressure-independent attribution to autophagy is therefore not established for any intervention in this literature. Conversely, the nonpressor Ang II design of Sugawara et al. illustrates how pressure and receptor signaling can be dissociated experimentally, and that design principle deserves wider adoption [97].

### 10.2. RAAS Blockade and the Aldosterone Axis: Recent Developments

Angiotensin-converting enzyme (ACE) inhibitors and angiotensin receptor blockers are established therapy in hypertensive CKD, and their benefit long predates any autophagy hypothesis. The mechanistic work reviewed here offers a coherent additional rationale: if chronic AT_1_R signaling impairs podocyte autophagic flux through calpain activation, AT_1_R blockade should preserve flux, and calpain becomes a downstream target addressable without further pressure reduction [78].

The aldosterone axis has become particularly relevant because therapeutic options have recently expanded. Finerenone is established in diabetic CKD and met its primary endpoint in the phase 3 FIND-CKD trial in nondiabetic CKD, improving eGFR slope in a population that included patients with hypertension-associated kidney disease [140]. Baxdrostat, a selective aldosterone synthase inhibitor, lowers blood pressure in patients with uncontrolled hypertension, including those with CKD, and received regulatory approval for hypertension in 2026 [141,142,143]. This approval establishes antihypertensive efficacy and should not be interpreted as evidence of renal outcome benefit; whether aldosterone synthase inhibition slows CKD progression is being tested separately, and no autophagy-related mechanism has been demonstrated for either agent.

A mechanistic distinction between MR antagonism and aldosterone synthase inhibition nevertheless remains of interest. Aldosterone can enhance podocyte autophagic flux through FOXO1 and RAB7 as a compensatory response [79]. MR blockade and suppression of aldosterone synthesis therefore need not have identical cellular consequences, even when both reduce blood pressure and albuminuria. This possibility is currently hypothetical, and autophagy should not be considered an established determinant of the clinical benefit of either drug class.

The apparently divergent experimental findings—aldosterone-enhanced podocyte flux in vitro [79] versus improvement of autophagy-associated markers with spironolactone in hypertensive or mechanically stressed systems [40,126] —may reflect the distinction between an acute compensatory response and the net consequences of chronic MR activation. Under the ACGEM framework, however, the relevant therapeutic studies generally lack the combination of dynamic in vivo flux assessment (A2), renal cell-type resolution (C2), and autophagy-specific causal perturbation (E/G) required to attribute protection specifically to autophagy. Their findings therefore support association rather than mechanistic attribution.

### 10.3. SGLT2 Inhibitors: A Plausible but Incompletely Tested Autophagy Mechanism

SGLT2 inhibitors reduce kidney disease progression across diabetic and nondiabetic CKD and are standard therapy in much of the population relevant to this review. Experimental studies, predominantly in diabetic renal models, have linked their effects to activation of AMPK and SIRT1, suppression of mTORC1 and maladaptive HIF-1α signaling, and improvement in autophagy-associated and mitochondrial phenotypes [144,145,146,147]. These observations provide biological plausibility but do not establish autophagy as the mechanism underlying clinical renoprotection.

Viewed through the ACGEM framework, much of this evidence relies on upstream signaling or static autophagy-associated measurements rather than cell-type-resolved in vivo flux. In addition, SGLT2 inhibitors have major nonautophagic actions, including restoration of tubuloglomerular feedback, reduction of proximal tubular workload, and hemodynamic effects. Thus, direct evidence that modulation of autophagic flux mediates their benefit specifically in hypertensive kidneys remains lacking.

A recent study by Matsui et al. is particularly relevant to the clearance hypothesis developed in Section 9. Empagliflozin reduced LRP2/megalin-mediated tubular albumin exposure, thereby lowering autophagic demand and preventing proximal tubular autophagic stagnation, with parallel reductions in lysosomal phospholipid accumulation, inflammation, and fibrosis [75]. The study combined in vivo assessment with inducible proximal-tubule-specific Lrp2 and Atg5 manipulation, providing strong cell-specific and causal evidence across several ACGEM dimensions. However, the models were high-fat-diet obesity and 5/6 nephrectomy rather than systemic hypertension, limiting disease-model relevance to hypertensive CKD. The study therefore supports the biological principle that reducing autophagic load can preserve productive flux, but it should not be treated as direct evidence for the same mechanism in hypertensive nephrosclerosis.

### 10.4. mTOR Inhibition: A Suggestive but Imperfect Discriminating Test

Rapamycin attenuates salt-induced hypertension and kidney injury in Dahl salt-sensitive rats [29,30], but autophagy was not directly measured in these studies. Their protective effects therefore provide signaling context rather than evidence that autophagy induction mediated renoprotection.

Interpretation is further complicated in podocytes. Sirolimus has been associated with de novo proteinuria and FSGS after transplantation and disrupts pathways required for podocyte integrity in experimental systems [135,136]. Moreover, basal podocyte autophagy is regulated predominantly through AMPK–ULK1 rather than canonical mTORC1 signaling, and the autophagic response to rapamycin changes with treatment duration [138]. Thus, adverse or neutral responses to mTORC1 inhibition cannot themselves establish a clearance-side lesion. As discussed in Section 9, direct flux reporters combined with lysosomal functional measurements provide a more informative way to discriminate initiation-limited from clearance-limited states.

### 10.5. Traditional Chinese Medicine and Botanical Compounds

Traditional Chinese medicines and plant-derived compounds constitute a notable part of the experimental literature linking renal protection to autophagy or mitophagy. Most of the mechanistically detailed evidence, however, comes from diabetic rather than hypertensive kidney disease. Recent examples include dendrobine, which was reported to enhance SIRT1/FOXO3a-associated mitophagy and reduce endothelial senescence in diabetic kidney disease; “Qi Nan” agarwood, which improved podocyte injury together with changes in EGFR-associated autophagy; DMDD isolated from *Averrhoa carambola*, which modulated endoplasmic-reticulum-stress–autophagy crosstalk in diabetic nephropathy; and Qufeng Tongluo decoction, which was studied in high-glucose/H_2_O_2_-injured podocytes [148,149,150,151]. These studies support the broader pharmacological tractability of autophagy and mitophagy in renal disease but cannot be regarded as direct therapeutic evidence for hypertension-attributed CKD because their upstream metabolic injury and disease context are fundamentally different.

A smaller literature directly addresses hypertensive renal injury. Qian Yang Yu Yin granule (QYYYG) ameliorated renal injury in spontaneously hypertensive rats and altered BECN1, LC3-II/I, and SQSTM1/p62 together with TRPC6–CaMKKβ–AMPK–mTOR signaling; complementary Ang II-treated podocyte experiments linked these changes to the same signaling axis [86]. Cordyceps cicadae reduced renal fibrosis and altered SIRT1-associated autophagy markers in SHR and Ang II-treated tubular-cell systems [34]. JiangyaTongluo decoction was associated with improved mitochondrial morphology, PINK1/PRKN-related mitophagy markers, and reduced tubulointerstitial fibrosis in hypertensive nephropathy [26].

These findings are potentially relevant but should be interpreted conservatively. QYYYG and Cordyceps cicadae are multicomponent preparations, the in vivo studies rely predominantly on static autophagy-associated measurements, and none demonstrates cell-type-resolved dynamic flux in the hypertensive kidney. The JiangyaTongluo and Cordyceps studies also share institutional and author lineage and therefore should not be counted as independent replication. Under the ACGEM framework, their relatively strong hypertension-model relevance (M) is not accompanied by equally strong autophagy measurement (A), cell-type resolution (C), genetic evidence (G), or autophagy-specific experimental causality (E).

Thus, the Chinese medicine literature reinforces the possibility that renal autophagy and mitophagy are pharmacologically modifiable in hypertensive injury, but it does not yet establish either pathway as the mediator of renoprotection. Future studies using defined active constituents, pressure-matched controls, cell-type-resolved flux reporters, and pathway-specific perturbation will be required to distinguish autophagy-dependent effects from the broad antioxidant, anti-inflammatory, metabolic, and hemodynamic actions of these compounds.

### 10.6. Metabolic, Nutritional, and Lifestyle Interventions

Exercise training reduced HIF-1α, autophagy-related markers, and fibrosis in SHRs [37], whereas a ketogenic diet reduced autophagy-related protein expression and aggravated injury [66]. Both interventions have broad metabolic and hemodynamic effects, and neither provides sufficient autophagy-specific causal evidence to attribute the renal phenotype to altered autophagic activity. They nevertheless demonstrate that the metabolic environment can modify the autophagy-associated phenotype of the hypertensive kidney.

### 10.7. What Would Make Translation Rational

These observations do not imply that autophagy is untargetable in hypertensive CKD. Rather, several unresolved questions currently prevent rational target selection. The desired direction of intervention may differ between podocytes and proximal tubules; the relevant disease stage remains undefined; no validated biomarker of renal autophagic flux exists for patient selection or target engagement; and, most importantly, it remains uncertain whether the limiting lesion lies predominantly at autophagosome formation or downstream clearance.

Resolving the formation-versus-clearance question with cell-type-resolved flux and lysosomal functional assays would therefore substantially refine therapeutic development. A translatable readout of renal autophagic or lysosomal activity would additionally be required to establish target engagement in humans. Until these mechanistic gaps are resolved, the most defensible clinical strategy remains to target established upstream drivers of hypertensive CKD with therapies of proven renal benefit, while treating autophagy and mitophagy modulation as candidate mechanisms to be tested rather than therapeutic effects that can be assumed.

## 11. Unresolved Questions and a Methodologic Roadmap

Progress in this field is limited less by the absence of hypotheses than by the measurements needed to distinguish among them. Table 5 summarizes a proposed minimum experimental standard based on the independent ACGEM dimensions and links each methodological element to the mechanistic question it can address.

Five questions appear particularly important.

1. Where is autophagic flux limited, and does this change over time?
  The central unresolved issue is whether chronic hypertensive stress limits autophagosome formation, lysosomal clearance, or both at different disease stages. Longitudinal studies using tandem LC3 reporters together with direct lysosomal functional assays in chronic Ang II or mineralocorticoid-salt models could simultaneously test the proposed formation-versus-clearance and “early adaptation, late failure” models described in Section 9.
2. How does autophagic and mitophagic flux change in specific renal cell types during hypertension in vivo?
  Direct longitudinal flux measurements in podocytes and tubular epithelial cells remain scarce. Cell-type-resolved reporter approaches are needed because whole-kidney measurements can obscure divergent responses among renal compartments.
3. Which mitophagy pathways operate in the hypertensive kidney?
  Reliance on PINK1 and PRKN abundance is insufficient, particularly because basal renal mitophagy can occur substantially independently of PINK1. Reporter-based assessment combined with pathway-specific perturbation will be required to distinguish PINK1/PRKN-dependent from receptor-mediated pathways.
4. How do vascular and epithelial autophagy interact during disease progression?
  Whether endothelial or vascular-cell dysfunction precedes and drives downstream podocyte and tubular autophagic abnormalities remains unresolved. Cell-specific genetic models will be required to establish the direction of causality between vascular injury, capillary rarefaction, and epithelial quality-control failure.
5. Does the autophagy phenotype differ among molecular subtypes of hypertension-attributed CKD?
  Human disease attributed clinically to hypertension is biologically heterogeneous. Genotype-stratified studies, including consideration of APOL1 where relevant, may help distinguish intrinsic endolysosomal abnormalities from mechanisms associated with classical hypertension-driven arterionephrosclerosis.

A major limitation common to these questions is the absence of direct pathological validation in human hypertensive nephrosclerosis. To our knowledge, cell-type-resolved autophagic flux or functional lysosomal abnormalities have not been demonstrated in biopsy-confirmed classical hypertensive nephrosclerosis. Initial studies of archived biopsy material using LC3, SQSTM1/p62, LAMP1, TFEB, and complementary lysosomal markers could establish whether the abnormalities predicted by experimental models are present in human disease. Such observations would remain descriptive without functional flux measurements, but they would provide an essential bridge between experimental models and human pathology.

The ACGEM framework also highlights why apparent convergence across studies should be interpreted cautiously. Studies may share high disease-model relevance (M) while remaining limited in autophagy measurement (A), cell-type resolution (C), genetic evidence (G), or autophagy-specific experimental causality (E). Shared authorship [26,34], divergent conclusions from a single laboratory [63,99], and extrapolation from nonhypertensive models to hypertensive contexts [28,97] further limit the extent to which apparently similar observations can be treated as independent mechanistic confirmation. Evaluating these dimensions separately is therefore more informative than assigning overall strength on the basis of the number of concordant studies.

## 12. Conclusions

Autophagy and mitophagy are plausible determinants of renal adaptation to chronic hypertensive stress, but the available evidence remains substantially narrower than is often implied. When assessed across the independent ACGEM dimensions, most studies rely on static autophagy-associated markers (A1), frequently in whole-kidney tissue with limited cell-type resolution (C0–1). Direct flux measurements (A2), cell-specific genetic manipulation (G1–2), and autophagy-specific causal perturbation (E1–2) remain uncommon, and high disease-model relevance (M2) does not compensate for limitations in these other dimensions.

The most informative podocyte studies combine cell-type resolution, dynamic assessment, and genetic perturbation and support a protective role for intact autophagic flux during chronic Ang II stress [78]. Tubular studies likewise suggest that maintaining or enhancing epithelial autophagic flux can limit injury under mineralocorticoid stress [31], but equivalent cell-specific genetic evidence remains limited. Rather than defining a single direction of autophagy change in hypertensive CKD, the current literature therefore supports a context-dependent response shaped by cell type, hypertensive stimulus, disease stage, and the component of the pathway being measured.

Interpretation is further complicated by the heterogeneity of clinically defined hypertensive nephrosclerosis. Biopsy-based studies demonstrate substantial diagnostic misclassification, while genetically and etiologically distinct disorders may coexist within CKD attributed to hypertension. Accordingly, findings from APOL1-associated nephropathy, CINAC, or proteinuric kidney disease cannot be considered direct evidence for classical hypertension-driven arterionephrosclerosis.

A further limitation of the field is its emphasis on autophagosome formation and other upstream markers, whereas lysosomal competence and terminal cargo clearance remain largely unexamined. Lysosomal abnormalities reported in APOL1-associated disease, CINAC, and proteinuric overload provide precedent for renal lysosomal vulnerability, but extrapolation from these distinct disorders to hypertensive nephrosclerosis remains speculative. No study has yet directly characterized lysosomal function in biopsy-confirmed human arterionephrosclerosis.

We therefore propose, as a testable working hypothesis rather than an established mechanism, that limited lysosomal clearance may become a bottleneck in a subset of hypertensive CKD. This model predicts that increasing autophagy initiation would not necessarily increase degradation when lysosomal capacity is limiting and could instead increase autophagosome burden. Resolving this possibility will require longitudinal, cell-type-resolved flux measurements together with direct assessment of lysosomal competence in hypertension-relevant models and human kidney tissue. Such studies will determine whether future autophagy-directed strategies should enhance initiation, restore clearance, or target specific molecular and cellular subtypes of hypertensive CKD.

## Figures and Tables

**Table 2:** Experimental models of hypertensive kidney disease and the strength of autophagy evidence in each.

Model	Dominant Pathogenic Stress	HTN-CKD Phenotype Reproduced	Best Available Autophagy Evidence (ACGEM)	Principal Interpretive Limitation
Ang II infusion	AT_1_R signaling plus pressure	Albuminuria, glomerular injury, tubulointerstitial fibrosis	A2/C2/G2/E2/M2 [78]	Direct cellular Ang II effects inseparable from pressure; lesions milder than human nephrosclerosis
SHR	Genetic, gradually developing hypertension	Chronic hypertension; variable, often mild fibrosis	A2/C1/G0/E1/M2 [37]	Advanced nephrosclerosis often absent; most studies are diet, exercise, or pharmacologic interventions
DOCA-salt/aldosterone-salt	MR activation, salt and volume loading, low renin	Vascular injury, glomerular and interstitial fibrosis	A2/C1/G1/E2/M2 [79]	Degree of mineralocorticoid-salt loading atypical of essential hypertension
2K1C	Endogenous RAAS activation plus unilateral ischemia	Renovascular hypertension; asymmetric bilateral pathology	N/A from current study set	Two kidneys sustain different insults; pooled analysis uninterpretable
Dahl salt-sensitive	Salt-sensitive hypertension, NOX4-derived H_2_O_2_	Albuminuria, inflammation, fibrosis	A1/C1/G0/E0/M2 [72]	Direct Dahl-SS evidence remains marker based; no rigorous autophagic-flux or autophagy-specific causal manipulation
5/6 nephrectomy	Nephron loss, hyperfiltration; hypertension secondary	CKD progression, proteinuria, fibrosis	A2/C2/G2/E2/M0 [75]	Hypertension is not the primary driver
L-NAME	Nitric oxide deficiency, endothelial dysfunction	Endothelial injury, glomerulosclerosis	N/A from current study set	Very little direct renal autophagy data

**Table 5:** Proposed minimum reporting standard and priority experiments for autophagy studies in hypertensive kidney disease.

Element	Minimum Requirement	Question Addressed
Flux, not markers	Tandem reporter (mRFP/mCherry-GFP-LC3), mt-Keima, or mito-QC; or lysosomal-inhibitor clamp with quantified LC3-II accumulation, in vivo where feasible	Is degradation genuinely increased or decreased?
Cell-type resolution	Cell-specific reporters or co-staining with lineage markers; avoid whole-kidney lysates as the sole readout	Which cell population is responsible?
Cell-type-specific genetics	Podocyte- or PTEC-specific deletion of *Atg5*, *Atg7*, or *Rb1cc1* in a hypertensive model; conditional and inducible where stage matters	Is autophagy required for the phenotype in that cell?
Longitudinal design	Flux measured at 3 or more time points spanning early and established disease in the same model	Does adaptation precede failure, and when?
Lysosomal competence (highest priority)	Cathepsin B/D activity, lysosomal pH, LAMP1 morphology, nuclear TFEB, measured alongside flux in the same tissue	Is the defect in formation or in degradation? Determines whether initiation enhancers help or harm
Mitophagy beyond PINK1/PRKN	Ratiometric mitophagy reporters; assessment of BNIP3, BNIP3L, FUNDC1, and PHB2 alongside PINK1/PRKN	Which mitophagy route operates in the hypertensive kidney?
Pressure control	Pressure-matched comparator arm, or a nonpressor design	Is the effect blood pressure-independent?
Provenance transparency	Explicit statement of shared authorship or institutional lineage with cited prior work	Is apparent convergence genuinely independent?
Human validation	Autophagy, mitophagy, and lysosomal markers in biopsy-confirmed hypertensive nephrosclerosis with spatial resolution; report *APOL1* genotype where ancestry makes it relevant	Does the mechanism operate in human disease, and in which molecular subtype?

## Data Availability

No new data were created or analyzed in this study. All data discussed are available in the cited primary publications.

## Supplementary Materials

The following supporting information can be downloaded at: https://www.mdpi.com/article/10.3390/life16091481/s1. Table S1: Literature search strategy and study eligibility criteria, including the PubMed search string, date of the final search, inclusion and exclusion criteria, and the study-selection process; Table S2: Study-by-study ACGEM grading of the 51 included publications, listing for each publication the experimental model, renal compartment or cell type examined, principal autophagy/mitophagy readouts, the assigned grade for each of the five ACGEM dimensions (autophagy/mitophagy measurement, cell-type resolution, genetic evidence, causal evidence, and model relevance), and the key interpretive limitations.

## Abbreviations

The following abbreviations are used in this manuscript: ACGEM, autophagy/mitophagy measurement, cell-type resolution, genetic evidence, causal evidence and model relevance; 2K1C, two-kidney one-clip; ACE, angiotensin-converting enzyme; AMPK, AMP-activated protein kinase; Ang II, angiotensin II; APOL1, apolipoprotein L1; AT_1_R, angiotensin II type 1 receptor; ATG, autophagy-related; BECN1, Beclin-1; CINAC, chronic interstitial nephritis in agricultural communities; CKD, chronic kidney disease; DOCA, deoxycorticosterone acetate; EMT, epithelial-mesenchymal transition; FSGS, focal segmental glomerulosclerosis; HIF-1α, hypoxia-inducible factor 1α; HTN-CKD, hypertensive chronic kidney disease; LAMP1, lysosome-associated membrane protein 1; LC3, microtubule-associated protein 1 light chain 3; MR, mineralocorticoid receptor; mtDNA, mitochondrial DNA; mTORC1, mechanistic target of rapamycin complex 1; NLRP3, NLR family pyrin domain containing 3; NOX4, NADPH oxidase 4; PGC-1α, peroxisome proliferator-activated receptor gamma coactivator 1α; PHB2, prohibitin 2; PINK1, PTEN-induced kinase 1; PRKN, parkin; PTEC, proximal tubular epithelial cell; RAAS, renin-angiotensin-aldosterone system; RAB7, Ras-related protein Rab-7a; ROS, reactive oxygen species; SGLT2, sodium-glucose cotransporter 2; SHR, spontaneously hypertensive rat; SQSTM1, sequestosome 1; STING, stimulator of interferon genes; TFEB, transcription factor EB; ULK1, unc-51 like autophagy activating kinase 1; VEGFR3, vascular endothelial growth factor receptor 3.
