# Supplementary material for: Autophagy and Mitophagy in Hypertensive Chronic Kidney Disease: Evidence Grading, Cell-Type Divergence, and a Working Lysosomal Hypothesis
Source: Life (Basel). 2026 Sep 4;16(9):1481. doi: 10.3390/life16091481 (PMC13608432; doi:10.3390/life16091481)
Supplement: Supplementary file 1 [file life-16-01481-s001.zip › life-4512936-supplementary.pdf]

**Supplementary Table S1.** Summary of the literature search and study-selection strategy.

| Items | Specification |
|---|---|
| **Date of search** | August 19, 2026 |
| **Database** | PubMed |
| **Search terms used** | Terms related to hypertension and hypertensive kidney disease (“hypertension,” “hypertensive nephropathy,” “hypertensive nephrosclerosis,” “angiotensin II,” “aldosterone,” “DOCA-salt,” “spontaneously hypertensive,” “Dahl salt-sensitive,” “two-kidney one-clip,” and “2K1C”) were combined with terms related to autophagy and mitophagy (“autophagy,” “autophagic flux,” “mitophagy,” “lysosome,” “LC3,” “SQSTM1,” “p62,” “PINK1,” and “Parkin”) and renal terms (“kidney,” “renal,” “podocyte,” and “tubular”). Search terms were restricted to the title/abstract fields.<br>**Search strategy:** (hypertension[Title/Abstract] OR "hypertensive nephropathy"[Title/Abstract] OR "hypertensive nephrosclerosis"[Title/Abstract] OR "angiotensin II"[Title/Abstract] OR aldosterone[Title/Abstract] OR "DOCA-salt"[Title/Abstract] OR "spontaneously hypertensive"[Title/Abstract] OR "Dahl salt-sensitive"[Title/Abstract] OR "two-kidney one-clip"[Title/Abstract] OR 2K1C[Title/Abstract]) AND (autophagy[Title/Abstract] OR "autophagic flux"[Title/Abstract] OR mitophagy[Title/Abstract] OR lysosome*[Title/Abstract] OR LC3[Title/Abstract] OR SQSTM1[Title/Abstract] OR p62[Title/Abstract] OR PINK1[Title/Abstract] OR Parkin[Title/Abstract]) AND (kidney[Title/Abstract] OR renal[Title/Abstract] OR podocyte*[Title/Abstract] OR tubul*[Title/Abstract]) |
| **Timeframe** | From database inception to August 19, 2026 |
| **Inclusion and exclusion criteria** | Primary experimental and human studies were considered relevant when they examined autophagy- or mitophagy-related signaling, markers, degradative flux, or pathway dependence in renal tissue or renal cell types under hypertensive or hypertension-relevant conditions. Studies using nonhypertensive experimental models were considered only when they addressed a mechanistic question directly relevant to the renal cell type or pathway under discussion and were identified separately as contextual evidence. Studies without kidney-related autophagy or mitophagy data were excluded from the primary evidence assessment. |
| **Selection process** | Titles and abstracts were screened for relevance, followed by full-text assessment of potentially relevant studies. Studies were selected according to their relevance to renal autophagy or mitophagy in hypertensive or hypertension-relevant conditions. Reference lists of relevant primary studies and reviews were additionally examined to identify studies pertinent to specific mechanisms discussed in the review. |
| **Evidence assessment** | Included studies were evaluated separately according to the type of autophagy or mitophagy measurement, cell-type resolution, and evidence for pathway dependence or causality, according to the criteria defined in Table 1. Model relevance was assessed separately from these evidence dimensions. |

**Supplementary Table S2.** Study-by-study ACGEM grading of autophagy evidence relevant to experimental hypertensive kidney disease

| Compartment | Stress axis / model | Study | Experimental system | Key autophagy/mitophagy evidence | A | C | G | E | M | Interpretation for review | Key caveat |
|---|---|---|---|---|---|---|---|---|---|---|---|
| Podocyte | Basal homeostasis | Hartleben et al., 2010 [14] | Podocyte-specific Atg5 KO mice | Constitutive autophagy; ubiquitinated/oxidized protein accumulation, ER stress, podocyte loss, glomerulosclerosis | **2** | **2** | **2** | **2** | **0** | Basal autophagy is essential for long-term podocyte homeostasis and stress resistance | Chloroquine-supported GFP-LC3/LC3-II turnover meets A2; podocyte-specific Atg5 deletion supports C2/G2/E2. Nonhypertensive comparator (M0). |
| Podocyte | Ang II, early | Yadav et al., 2010 [81] | Cultured podocytes + Ang II | LC3, autophagosomes, ROS-associated autophagy | 1 | 1 | 0 | 0 | 1 | Ang II induces an early stress-associated autophagy response | Static readouts; no rigorous flux or chronic HTN |
| Podocyte | Ang II, pharmacologic | Wang et al., 2016 (sinomenine) | Cultured podocytes + Ang II ± sinomenine | LC3B, TEM autophagosomes/autolysosomes, ROS; NADPH oxidase p47-phox membrane translocation; DPI | **1** | **1** | **0** | **0** | **1** | Sinomenine attenuates Ang II-associated autophagy by suppressing p47-phox-dependent ROS generation | No dynamic flux; pharmacologic evidence only; reduced autophagosome abundance cannot establish reduced flux |
| Podocyte | Ang II, pharmacologic | Mao et al., 2016 (Rg1) [83] | MPC5 podocytes + Ang II ± ginsenoside Rg1 | Autophagosomes, LC3-II, BECN1; AMPK/GSK3β/mTOR-P70S6K signaling | 1 | 1 | 0 | 0 | 1 | Rg1 suppresses Ang II-associated autophagy markers, but the functional significance of this suppression is unresolved | No flux and no demonstration that autophagy inhibition mediates protection |
| Podocyte | Ang II, early | Gao et al., 2017 [84] | Cultured podocytes + Ang II | ROS, TRPC6-mediated $Ca^{2+}$ influx, LC3-II/I↑, BECN1↑, p62↓; rapamycin/3-MA | 1 | 1 | 0 | 1 | 1 | ROS–TRPC6–$Ca^{2+}$ signaling induces an autophagy-associated response; pharmacologic enhancement improves cell viability | No dynamic flux; pharmacologic causality only |
| Podocyte | Ang II, early | Seong et al., 2019 [85] | Mouse podocytes + Ang II | LC3A/B-II, BECN1, ATG5 siRNA, apoptosis | **1** | **1** | **1** | **2** | **1** | Autophagy precedes apoptosis and appears to buffer acute Ang II injury | Short-term in vitro model; no dynamic flux |
| Podocyte | Ang II, chronic | Bensaada et al., 2021 [78] | Chronic Ang II hypertension; podocyte-specific Atg5 KO; calpastatin TG | Dynamic autophagy/flux assessment; calpain–calpastatin axis; podocytopathy and albuminuria | **2** | **2** | **2** | **2** | **2** | Chronic Ang II activates calpain and impairs podocyte autophagic flux; preserved autophagy is strongly protective | Chronic Ang II hypertensive kidney disease with podocyte-specific Atg5 deletion; cell-specific in vivo genetic evidence supports C2/G2/E2. |
| Podocyte | Ang II / SHR, therapeutic | Ma et al., 2023 (QYYYG) [86] | SHR + Ang II-treated rat podocytes; TRPC6 overexpression | GFP-LC3 puncta, LC3-II/I, BECN1, p62; TRPC6–CaMKKβ–AMPK–mTOR | **1** | **1** | **0** | **1** | **2** | In a disease-relevant SHR model, QYYYG reduces Ang II-associated autophagy markers together with podocyte injury | No flux; TRPC6 manipulation establishes upstream signaling but not autophagy→injury causality |
| Podocyte | Ang II, therapeutic | Liu et al., 2026 (YGYSG) [87] | Ang II-stimulated MPC-5 podocytes ± YGYSG medicated serum | TEM, LC3-II/I, p62, BECN1; PI3K/AKT/mTOR; 3-MA | **1** | **1** | **0** | **1** | **1** | YGYSG reverses Ang II-associated autophagy-marker changes, consistent with PI3K/AKT/mTOR-linked modulation | No dynamic flux; in vitro only; autophagosome accumulation cannot distinguish formation from impaired clearance |

| | | | | | | | | | | | |
|---|---|---|---|---|---|---|---|---|---|---|---|
| Podocyte | Aldosterone/MR | Mao et al., 2014 [89] | MPC5 podocytes + aldosterone ± Rg1/NAC/CQ | LC3-II, BECN1, autophagosomes; CQ-supported accumulation; ROS | 2 | 1 | 0 | 0 | 1 | Aldosterone induces an early ROS-dependent autophagic response; Rg1 reduces upstream oxidative stress and autophagic demand | In vitro only; does not establish whether autophagy itself is protective or harmful |
| Podocyte | Aldosterone/MR | Yuan et al., 2015 [90] | Aldosterone-infused mice + cultured podocytes | ROS, ER stress/CHOP, LC3-associated autophagy; pharmacologic autophagy inhibition | 1 | 1 | 0 | 1 | 1 | ER stress triggers apoptosis together with a compensatory autophagic response; autophagy inhibition increases apoptosis | No dynamic flux; short aldosterone model without established chronic HTN phenotype |
| Podocyte | Aldosterone/MR | Bai et al., 2016 [91] | MPC5 podocytes + aldosterone | Tandem mRFP-GFP-LC3, TEM, LC3/p62; 3-MA and Atg5 siRNA; nephrin/apoptosis | 2 | 1 | 1 | 2 | 1 | Strong evidence that early ROS-induced autophagic flux is an adaptive response delaying aldosterone-induced injury | Cell-culture model only; no chronic hypertension |
| Podocyte | Aldosterone/MR | Wang et al., 2016 [79] | Cultured podocytes + aldosterone; Aldo/salt hypertensive rats | CQ-supported LC3 flux, p62; FOXO1/RAB7, Ac-FOXO1–ATG7; Atg7 siRNA; RP/CQ/3-MA | 2 | 1 | 1 | 2 | 2 | FOXO1–RAB7 and Ac-FOXO1–ATG7 signaling sustains protective podocyte autophagy during mineralocorticoid stress | In vivo autophagy assessed largely at glomerular rather than podocyte-specific level |
| Podocyte | Mechanical stress | Li et al.[40] | Human podocytes + stretch ± spironolactone | LC3 puncta, ATG5, MR, PI3K/AKT/mTOR, adhesion | **1** | **1** | **0** | **1** | **1** | Mechanical stress may alter MR–mTOR-linked podocyte autophagy and adhesion | Diabetic/mechanical-stress context; no HTN animal model or flux |
| Podocyte | Mitophagy comparator | Jiang et al., 2020 [92] | Palmitic acid-treated podocytes | PINK1/PRKN, mitochondrial ROS, apoptosis | 1 | 1 | 0 | 0 | 0 | PINK1/Parkin-associated mitophagy may limit lipotoxic podocyte injury | Lipotoxic, nonhypertensive model |
| Podocyte | Mitophagy comparator | Li et al., 2017 [93] | STZ diabetic mice + high-glucose podocytes | FOXO1, PINK1/PRKN, mitochondrial morphology | 1 | 1 | 0 | 0 | 0 | FOXO1-associated PINK1/Parkin signaling supports mitochondrial quality control in podocytes | Diabetic rather than hypertensive model |
| Tubule | Ang II, adaptive | Sugawara et al., 2021 [97] | Non-pressor Ang II in vivo; NRK-52E | LC3-II, autophagosomes, ULK1, losartan | **1** | **1** | **0** | **1** | **1** | AT1R–ULK1 signaling induces adaptive tubular autophagy and improves subsequent I/R tolerance | Proximal-tubule localization/defined renal cells support C1, not C2. Ang II preconditioning co-modified autophagy readout and subsequent renal injury, supporting E1. |
| Tubule | Ang II, anti-inflammatory | Peng et al., 2019 [98] | Ang II-treated HK-2 and primary PTECs | ATG5 manipulation, LC3, NF-κB, cytokines | 1 | 1 | 1 | 2 | 1 | ATG5-dependent autophagy restrains NF-κB activation and inflammatory cytokine production | Predominantly in vitro; in vivo validation used a nonhypertensive injury model rather than chronic Ang II hypertension |
| Tubule | Ang II, mitophagy | Xu et al., 2019 [99] | Ang II-treated tubular epithelial cells | LC3, CQ, PHB2 KD/OE, mitochondrial–lysosomal colocalization, NLRP3 | 1 | 1 | 1 | 2 | 1 | PHB2-mediated mitophagy limits mitochondrial dysfunction, NLRP3 activation, and tubular cell injury | In vitro only; no dedicated dynamic mitophagy-flux reporter or hypertensive animal model |

| | | | | | | | | | | |
|---|---|---|---|---|---|---|---|---|---|---|
| Whole kidney / mixed | Chronic Ang II | Shen et al., 2018 [63] | Ang II-infused mice ± vitamin $D_3$ | BECN1, LC3-II/LC3-I, mitochondrial dysfunction, fibrosis | **1** | **0** | **0** | **1** | **2** | Autophagy-associated markers increase with Ang II injury and decline together with injury after vitamin $D_3$ treatment | Whole-kidney static markers; flux not assessed; vitamin D is pleiotropic |
| Whole kidney / mixed | Chronic Ang II / TLR4 | Majumder et al., 2022 [64] | 4-week Ang II; TLR4-mutant mice | ATG3/7/12, BECN1, LC3, p62 | **1** | **0** | **0** | **1** | **2** | TLR4 mutation preserves autophagy-associated proteins and attenuates Ang II renal injury | TLR4 is an upstream regulator rather than an autophagy/mitophagy component; therefore G0. Mutation co-modified autophagy markers and renal phenotype, supporting E1. |
| Tubule | SHR / HIF-1α-mediated autophagy | Li et al., 2025 [37] | SHR ± 10-week MICT/HIIT; Ang II-treated HK-2 with HIF-1α inhibition/overexpression | Renal ATG5, LC3B-II, p62; HIF-1α; AKT/mTOR; CQ lysosomal blockade; HIF-1α lentiviral overexpression | 2 | 1 | 0 | 1 | 2 | MICT improves hypertensive renal dysfunction and fibrosis while reducing HIF-1α-associated excessive autophagy; HIF-1α manipulation and CQ support a mechanistic link in tubular cells. | Dynamic flux was interrogated mainly in HK-2 cells rather than in vivo; genetic manipulation targets upstream HIF-1α rather than core autophagy machinery; exercise has pleiotropic effects. |
| Whole kidney / mixed | Ang II / exercise | Baek et al., 2026 [65] | Chronic Ang II mice ± treadmill exercise | LC3-II/I, p62, ATG3/5/7 | **1** | **0** | **0** | **1** | **2** | Exercise normalizes Ang II-associated autophagy-marker dysregulation together with renal injury | Static whole-kidney markers; no lysosomal blockade or reporter-based flux |
| Tubule | Ang II / PTPN1 | Zhao et al., 2025 [100] | Ang II-infused mice; Ang II-treated tubular cells | LC3, BECN1, p62, PTPN1–AMPK/mTOR | **1** | **1** | **0** | **1** | **2** | PTPN1-linked signaling is associated with altered tubular autophagy-related activity and renal injury | No autophagy-specific perturbation or dynamic flux; renal protection occurred without BP reduction |
| Tubule | Aldosterone / ROS | Wang et al., 2015 [109] | Aldosterone-treated NRK-52E ± NAC/Rg1 | LC3-II, BECN1, autophagosomes, ROS, AMPK/mTOR | 1 | 1 | 0 | 0 | 1 | Aldosterone induces ROS-associated autophagy-related responses in tubular cells | Cell-only model; productive versus impaired flux and protective versus maladaptive significance remain unresolved |
| Tubule | Aldosterone / senescence | Cao et al., 2021 [102] | Aldosterone-treated PTECs; UNx + aldosterone rats | LC3/p62, CQ, TEM, NOX4, ROS, p21 | **2** | **1** | **0** | **1** | **1** | Productive autophagy limits oxidative stress and tubular senescence | Hypertension/BP phenotype not directly established; autophagy causality is primarily pharmacologic |
| Tubule | Aldosterone / DOCA-salt | Li et al., 2021 (DOCA-salt) [31] | DOCA-salt mice; aldosterone-treated HK-2 | Tandem RFP-GFP-LC3, AMPK/ULK1, CQ, EMT | 2 | 1 | 0 | 1 | 2 | Effective epithelial autophagic flux protects against EMT and renal fibrosis | Strong flux evidence but autophagy causality relies mainly on pharmacologic interventions rather than genetic manipulation |
| Tubule | SHR / oxidative stress | Cai et al., 2021/2022 (Cordyceps Cicadae) [34] | SHR kidneys; Ang II-treated tubular cells | LC3-II, BECN1, p62, CQ/rapamycin, SIRT1/FOXO3a | 1 | 1 | 0 | 1 | 2 | ROS-associated autophagy dysregulation accompanies hypertensive renal fibrosis | Reporter/genetic flux validation limited; multicomponent intervention complicates attribution |
| Whole kidney / mixed | SHR / mitophagy | Zhao et al., 2024 [26] | SHR hypertensive nephropathy | PINK1, PARKIN, BECN1, p62, TEM, SIRT1/PGC-1α | **1** | **0** | **0** | **1** | **2** | Reduced PINK1/PARKIN-associated mitophagy markers accompany SHR nephropathy | Expression/TEM-based inference; no direct mitophagy |

| | | | | | | | | | | | |
|---|---|---|---|---|---|---|---|---|---|---|---|
| | | | | | | | | | | | flux or autophagy-specific causal manipulation |
| Tubule | Salt-sensitive HTN | Takakura et al., 2017 [72] | Dahl salt-sensitive rats ± Cordyceps militaris | LC3-II/I, p62, cathepsin D, tubular LC3, mitochondrial proteins | **1** | **1** | **0** | **1** | **2** | Tubular autophagy-associated and mitochondrial adaptations occur during salt-sensitive hypertensive kidney injury | Tubular localization was histologic/IHC (C1, not C2). Cordyceps modified autophagy-associated markers together with renal histology/survival (E1). |
| Tubule | High salt / UCP2–ROS | Forte et al., 2021 [105] | High-salt renal PTECs; UCP2 KD/OE | Bafilomycin flux, tandem mRFP-GFP-LC3, LC3/ATG7, PARKIN, TAT-Beclin1 rescue | **2** | **1** | **0** | **2** | **1** | UCP2 sustains adaptive autophagic flux and limits ROS-mediated injury under high-salt stress | UCP2 genetic manipulation is upstream of autophagy and does not qualify for G under the strict rubric. High-salt renal-cell context is indirect (M1); direct autophagy rescue supports E2. |
| Tubule | D5R / mitochondrial ROS | Lee et al., 2021 [106] | Human RPT cells; D5R cells; Drd5−/− mice | LC3-II, ATG5, CQ, spautin-1, mitochondrial ROS | 2 | 1 | 0 | 1 | 0 | D5R-induced autophagy contributes to control of mitochondrial ROS | Mechanistically relevant to BP regulation but not a direct hypertensive renal-injury model |
| Tubule | Ang II / NL-SDT | Liu et al., 2025 [101] | 4-week Ang II hypertensive mice; TGF-β1-treated NRK-52E cells | LC3B, LC3 puncta, TEM autophagosomes; PI3K/AKT/mTORC1; CQ, 3-MA, rapamycin; NAC | 1 | 1 | 0 | 1 | 2 | NL-SDT activates a ROS–PI3K/AKT/mTORC1-associated autophagy response and attenuates hypertensive renal fibrosis and tubular EMT. | No rigorous reporter-based dynamic flux assay; autophagy causality relies on pharmacologic modulators with limited specificity; in vitro fibrosis model uses TGF-β1 rather than Ang II. |
| Tubule | Ang II / mitophagy–senescence | Liu et al., 2020 [28] | Ang II-treated NRK-52E; UUO rats | PINK1/PARKIN, LC3/p62, mitophagy dye, EX527/Mdivi-1 | 1 | 1 | 0 | 1 | 1 | SIRT1–PINK1/PARKIN-associated mitophagy limits tubular senescence | Ang II evidence is primarily cellular; in vivo validation uses UUO rather than hypertension |
| Tubule | Prolonged Ang II / mitophagy | Wu et al., 2025 [27] | Ang II mice; Ang II-treated HK-2; HSPA1L manipulation | Mitochondrial PARKIN/LC3/p62, TEM, colocalization | **1** | **1** | **0** | **1** | **2** | Mitophagy-associated responses rise early but become impaired during prolonged Ang II exposure; VEGFR3/HSPA1L restores the pathway | VEGFR3/HSPA1L are upstream regulators of PARKIN-dependent mitophagy, not direct autophagy/mitophagy components (G0). Perturbations changed mitophagy readouts and injury without definitive pathway-component mediation (E1). |

| | | | | | | | | | | | |
|---|---|---|---|---|---|---|---|---|---|---|---|
| Tubule | Reduced nephron mass / cargo overload | Matsui et al., 2025 [75] | HFD/5/6 Nx; inducible PTEC-specific genetic models | In vivo CQ flux, GFP-LC3, starvation reserve, lysosomal burden | **2** | **2** | **2** | **2** | **0** | Chronic cargo load can exceed tubular autophagic/lysosomal capacity | Inducible proximal-tubule-specific Atg5 manipulation supports C2/G2; lysosomal-clamp flux supports A2 and phenotype dependence supports E2. |
| Tubule | 5/6 nephrectomy / IGF-1–PI3K/Akt/mTOR | Li et al., 2022 (HeidihuangWan)[76] | 5/6 nephrectomized Wistar rats ± HDHW; IGF-1R inhibitor JB1 | BECN1, LC3-II/I; IGF-1/IGF-1R–PI3K/Akt/mTOR; renal fibrosis markers | **1** | **0** | **0** | **1** | **0** | HDHW reduces BECN1/LC3-II-associated autophagy markers while improving fibrosis through an IGF-1/PI3K/Akt/mTOR-linked pathway. | 5/6 nephrectomy is a nonhypertensive reduced-nephron-mass CKD model; static whole-kidney markers do not establish flux, and JB1 targets upstream IGF-1R rather than autophagy itself. |
| Tubule | 5/6 Nx + DOCA | Ko et al., 2023 [77] | 5/6 Nx + DOCA-salt ± dapagliflozin/sacubitril–valsartan | LC3B-II/I, ATG5, BECN1, PINK1/PARKIN | **1** | **0** | **0** | **1** | **2** | Treatment normalizes autophagy/mitophagy-associated markers together with improvement in renal injury | 5/6 nephrectomy combined with DOCA-salt produced an established hypertensive kidney injury model, so M2. Therapy co-modified autophagy/mitophagy markers and renal phenotype (E1). |
| VSMC / vascular | Hypertension / cyclic stretch | Li et al., 2021 (VSMC/cyclic stretch) [118] | Abdominal aortic coarctation hypertensive rats; primary rat VSMCs exposed to 15% cyclic stretch | LC3-II, p62 and ATG proteins; LC3 puncta; bafilomycin A1/CQ blockade; rapamycin; lamin A/C and emerin siRNA; in vivo CQ | **2** | **1** | **0** | **1** | **1** | Pathological mechanical stretch activates VSMC autophagy through nuclear-envelope protein–mTOR signaling, and autophagy supports VSMC proliferation/vascular remodeling. | VSMC specificity derives from culture/tissue localization rather than cell-type-restricted in vivo manipulation (C1). Pharmacologic autophagy perturbation supports E1; vascular hypertension model is indirect for hypertensive CKD (M1). |
| *VSMC / vascular* | *Vascular calcification* | *Yu et al., 2024 [122]* | *HFD + vitamin D3 rat vascular calcification; H2O2-treated A7r5 VSMCs* | *Autophagy-related proteins; SIRT1/CXCR4 signaling; SIRT1 and CXCR4 siRNA; chloroquine* | *1* | *1* | *0* | *1* | *0* | *Luteolin-associated autophagy contributes to suppression of VSMC osteogenic transformation through SIRT1/CXCR4 signaling.* | *Vascular calcification model is not hypertensive kidney disease; no rigorous dynamic autophagic-flux assay.* |
| Whole kidney / mixed | Chronic Ang II | Liu et al., 2021 (luteolin) | 4-week Ang II-infused ApoE−/− mice ± luteolin | Whole-kidney LC3 and p62 with renal histology, collagen and inflammatory markers | **1** | **0** | **0** | **1** | **2** | Luteolin increases autophagy-associated markers while attenuating Ang II-associated renal inflammation and fibrosis. | Static whole-kidney markers only; no autophagy-specific perturbation or flux assay; ApoE deficiency adds a nonstandard disease background. |

| | | | | | | | | | | | |
|---|---|---|---|---|---|---|---|---|---|---|---|
| Whole kidney / mixed | High-salt hypertension | Ding et al., 2022 (calcitriol) | 8% high-salt hypertensive rats ± calcitriol/losartan; HK-2 cells with GLIS2 siRNA | Renal LC3-II/I and p62; p-AMPK/p-mTOR; transcriptomics; GLIS2 knockdown in HK-2 | **1** | **0** | **0** | **1** | **2** | Calcitriol-associated renal protection accompanies AMPK/mTOR-linked autophagy-marker changes and restoration of GLIS2. | GLIS2 siRNA targets an upstream transcriptional regulator rather than an autophagy/mitophagy component (G0). Calcitriol co-modified markers and renal injury/fibrosis (E1). |
| Mesangial cell | Ang II / proliferation | Ding et al., 2020 (calycosin)[123] | Human glomerular mesangial cells treated with Ang II ± calycosin | LC3, BECN1 and p62 by western blot/immunocytochemistry; ERK signaling; proliferation and apoptosis | **1** | **1** | **0** | **1** | **1** | Calycosin reduces Ang II-associated autophagy markers together with mesangial-cell proliferation and ERK activation. | Cell-only model with static autophagy readouts; no autophagy-specific perturbation, so mediation by autophagy is not established. |
| Mesangial cell | Ang II / redox signaling | Fan et al., 2025 (carnosol) [125] | Ang II-infused mice; Ang II-treated SV40-MES13 glomerular mesangial cells | p62 phosphorylation and KEAP1 degradation; CQ, bafilomycin A1 and 3-MA; p62 siRNA; mTOR inhibition; NRF2/HO1 | 1 | 1 | 1 | 2 | 2 | Carnosol promotes p62-dependent autophagic KEAP1 degradation and NRF2 antioxidant signaling during Ang II renal injury. | Mechanistic experiments define selective p62/KEAP1 cargo handling rather than global autophagic flux; most causal dissection is cell-based. |
| Whole kidney / mixed | Dahl salt-sensitive / Mlycd deficiency | Kravtsova et al., 2026 [73] | Dahl SS Mlycd+/− rats; baseline and high-salt challenge; kidney proteomics/metabolomics | Autophagy/mTOR pathway enrichment with altered abundance of Vps34/Pik3c3, Atg9, Vps16 and related proteins | 1 | 0 | 0 | 0 | 2 | Partial Mlycd deficiency remodels autophagy-associated and metabolic machinery in a salt-sensitive hypertensive background. | Proteomic association only; no flux or autophagy-specific perturbation, and Mlycd deficiency does not establish hypertension-driven autophagic impairment. |
| Mesangial cell | Ang II / senescence | Yang et al., 2019 [124] | Human glomerular mesangial cells treated with Ang II | LC3-II/p62, TEM autophagic vacuoles, tandem GFP-RFP-LC3; 3-MA/rapamycin; STAT3 inhibitor and STAT3 siRNA | **2** | **1** | **0** | **1** | **1** | STAT3/mTOR-regulated autophagy promotes Ang II-induced mesangial-cell senescence; pharmacologic inhibition of autophagy attenuates senescence. | Tandem GFP-RFP-LC3 supports A2. STAT3 siRNA is upstream and does not qualify for G; pharmacologic autophagy perturbation supports E1 rather than definitive E2. |
| Distal tubule | Aldosterone / protein aggregation | Cheema et al., 2014 [103] | 7-day aldosterone-infused rats ± spironolactone; DCT/CNT-resolved renal analysis | LC3 immunoreactivity and electron microscopy; p62/HDAC6/ataxin-3 aggresome machinery; protein aggregates | **1** | **1** | **0** | **0** | **1** | Aldosterone induces distal-tubular protein aggregation without a corresponding aggresome/autophagosome response, suggesting limited degradative adaptation. | DCT/CNT localization by histology supports C1, not C2; no direct causal autophagy manipulation tied to the renal phenotype (E0). |
| Whole kidney / mixed | Aldosterone-salt / GSK-3β | Zhang et al., 2018 (SB-216763) [68] | 4-week aldosterone-salt hypertensive Wistar rats ± GSK-3β inhibitor SB-216763 ± 3-MA | Renal LC3-II, p62 degradation and LC3 puncta; 3-MA; tubulointerstitial injury and fibrosis | 1 | 0 | 0 | 1 | 2 | GSK-3β inhibition enhances autophagy-associated activity and protects against aldosterone-induced renal inflammation, fibrosis, and tubulointerstitial injury; 3-MA partially reverses renoprotection. | Autophagy assessment is static rather than a rigorous lysosomal-blockade flux assay; 3-MA is nonspecific and no genetic autophagy manipulation was performed. |

| | | | | | | | | | | |
|---|---|---|---|---|---|---|---|---|---|---|
| *Whole kidney / mixed* | *SHR / ketogenic diet* | *Jia et al., 2021 [66]* | *Spontaneously hypertensive rats and Wistar controls ± 4-week ketogenic diet* | *Renal cortex/medulla LC3-II and ULK by western blot; renal function and Masson fibrosis* | ***1*** | ***0*** | ***0*** | ***1*** | **2** | *Ketogenic diet reduces renal autophagy-associated protein levels in SHR while worsening metabolic disturbance, RAAS activation, blood pressure, and renal parenchymal dysfunction.* | *Only static LC3-II/ULK abundance was measured; no p62, lysosomal blockade, reporter-based flux, genetic manipulation, or autophagy-specific perturbation.* |
| Whole kidney / mixed | DOCA-salt / PI3K-Akt-mTOR | Ma et al., 2012 | Uninephrectomized DOCA-salt hypertensive rats | Renal p-PI3K, p-Akt and p-mTOR with inflammatory, fibrotic and apoptotic markers; no direct autophagy readout | 0 | 0 | 0 | 0 | 2 | DOCA-salt hypertension activates renal PI3K/Akt/mTOR signaling together with inflammation, fibrosis, and apoptosis. | Autophagy was not directly measured; mTORC1 signaling provides mechanistic context only and cannot establish the direction or magnitude of autophagic activity. |
| Whole kidney / mixed | Dahl salt-sensitive / NOX4-H2O2-mTORC1 | Kumar et al., 2020 [30] | Dahl SS and SS-Nox4−/− rats on high salt ± rapamycin; NRK-52E H2O2 experiments | NOX4 genetic deletion; renal mTORC1 readouts; rapamycin; BP and renal injury; no direct autophagy measurement | **0** | **0** | **0** | **0** | **2** | NOX4-derived H2O2 is an upstream activator of renal mTORC1 that contributes to salt-sensitive hypertension and kidney injury. | NOX4 knockout is upstream of mTORC1 and no direct autophagy/mitophagy readout or pathway-component genetic manipulation was performed; A0/G0/E0. |
| | | | | | | | | | | | |
| | | | | | | | | | | | |

*ACGEM dimensions are graded independently and should not be summed into a single composite score. A/C/G/E characterize autophagy evidence; M reflects direct relevance of the experimental model to hypertensive kidney disease. mTOR-pathway studies without direct autophagy measurements are therefore scored A0 even when genetic or disease-model evidence is strong. The unit of analysis is the individual publication. Each eligible publication is counted once and assigned the highest unequivocally demonstrated grade within each ACGEM dimension. Multiple experimental arms, treatment groups, or assays within the same publication are not counted as separate studies. A, C, G, E, and M are evaluated independently and are not summed into a composite score. A total of 51 publications were included in the study-by-study ACGEM assessment. The grading criteria applied to each ACGEM dimension, together with the interpretive purpose and the principal caveat of each dimension, are given in Table 1 of the main text.*
